# A soluble molecular variant of the semiconducting silicondiselenide†
†Electronic supplementary information (ESI) available: Syntheses, NMR, UV-vis, Raman spectra, crystallographic table, and theoretical details. CCDC 926618, 927696, 948799, 983863, 1060365. For ESI and crystallographic data in CIF or other electronic format see DOI: 10.1039/c5sc01516b



**DOI:** 10.1039/c5sc01516b

**Published:** 2015-06-18

**Authors:** Kartik Chandra Mondal, Sudipta Roy, Birger Dittrich, Bholanath Maity, Sayan Dutta, Debasis Koley, Suresh Kumar Vasa, Rasmus Linser, Sebastian Dechert, Herbert W. Roesky

**Affiliations:** a Institut für Anorganische Chemie , Georg-August-Universität , 37077 Göttingen , Germany . Email: hroesky@gwdg.de; b Institut für Anorganische und Angewandte Chemie , Universität Hamburg , 20146 Hamburg , Germany , Raum AC 15c (Erdgeschoss); c Dept. of Chemical Sciences , Indian Institute of Science Education and Research (IISER) Kolkata , Mohanpur – 741246 , India; d Max-Planck-Institut für Biophysikalische Chemie , Abtl. NMR-basierte Strukturbiologie , 37077 Göttingen , Germany

## Abstract

Silicondiselenide is a semiconductor and exists as an insoluble polymer (SiSe_2_)_*n*_ which is prepared by reacting elemental silicon with selenium powder in the temperature range of 400–850 °C. Herein, we report on the synthesis, isolation, and characterization of carbene stabilized molecular silicondiselenide in the form of (cAAC)_2_Si_2_Se_4_ (**3**) [cAAC = cyclic alkyl(amino)carbene]. **3** is synthesized *via* reaction of diatomic silicon(0) compound (cAAC)_2_Si_2_ (**2**) with black selenium powder at –78 °C to room temperature. The intensely orange colored compound **3** is soluble in polar organic solvents and stable at room temperature for a month under an inert atmosphere. **3** decomposes above 245 °C. The molecular structure of **3** has been confirmed by X-ray single crystal diffraction. It is also characterized by UV-vis, IR, Raman spectroscopy and mass spectrometry. The stability, bonding, and electron density distributions of **3** have been studied by theoretical calculations.

## Abstract 2

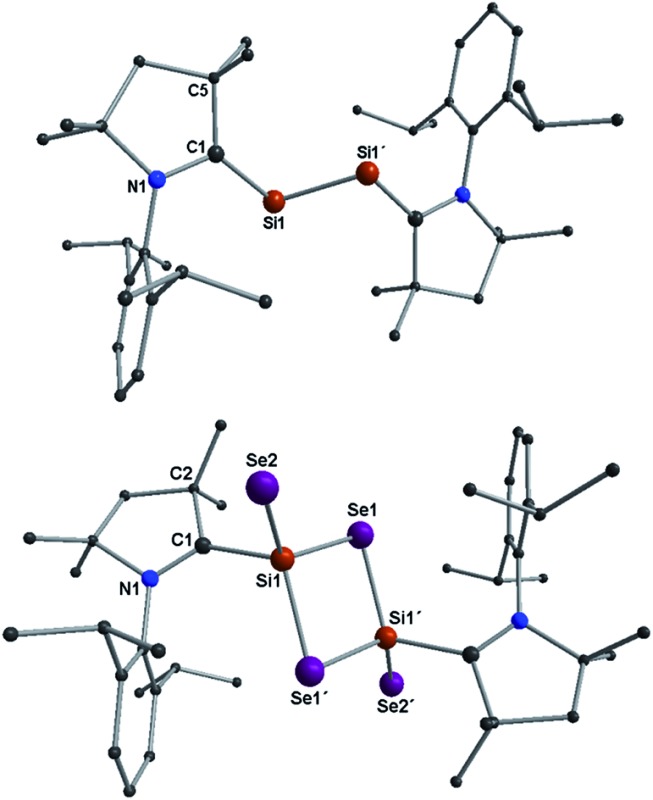
Silicondiselenide is a semiconductor and exists as an insoluble polymer (SiSe_2_)_*n*_ which is prepared by reacting elemental silicon with selenium powder in the temperature range of 400–850 °C.

## Introduction

**Scheme 1:**
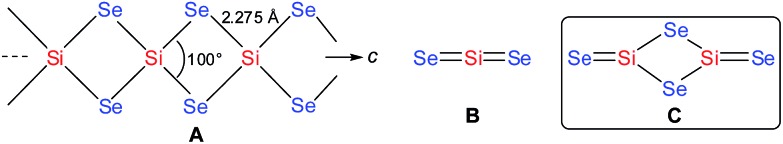
Schematic representation of some forms of SiSe_2_.

The synthesis of silicon diselenide (SiSe_2_; **A** in Scheme 1) was first reported by P. Sabatier in 1891.^1^ Six decades later crystalline SiSe_2_ was prepared by Weiss *et al.* ^2^ who reported the Bravais lattice of the crystal structure of SiSe_2_ as body-centered orthorhombic. Structural analysis showed that it crystallizes in space group *Ibam*.^3a–b^ SiSe_2_ has a unique crystalline structure which consists of infinite nonintersecting chains of edge-sharing tetrahedra (**A**) as shown in Scheme 1.^3a–b^ The structural scenario of amorphous SiSe_2_ is more complicated,^3*c*^ where the Si atoms are linked *via* intra- and inter-tetrahedral bonds connected by Se atoms. The predominant structural motif of amorphous SiSe_2_ follows the sequence involving both corner- and edge-sharing connections,^3*c*^ which are importantly more frequent than those constructed exclusively by edge-sharing Si atoms in crystalline SiSe_2_.^3a–b^

Several modified synthesis routes have been developed in quest of the physical properties of SiSe_2_ in a single phase. Large red rods of SiSe_2_ have been grown for 4–7 days inside a quartz tube at 1000 °C under high vacuum placed in a furnace.^4–6^ They are highly moisture sensitive and undergo hydrolysis on the surface to produce silicon- and selenium dioxide.^4^ The glass transition temperature and crystallization temperature of SiSe_2_ are 460 and 610 °C, respectively.^5^ Further studies showed that SiSe_2_ can exist as three different polymorphs depending upon the temperature (400–850 °C) of preparation.^6^ Crystalline SiSe_2_ is considered to be a semiconductor^4,7a–b^ and has a huge potential to be used in solar cells.^7*c*^

**Scheme 2:**
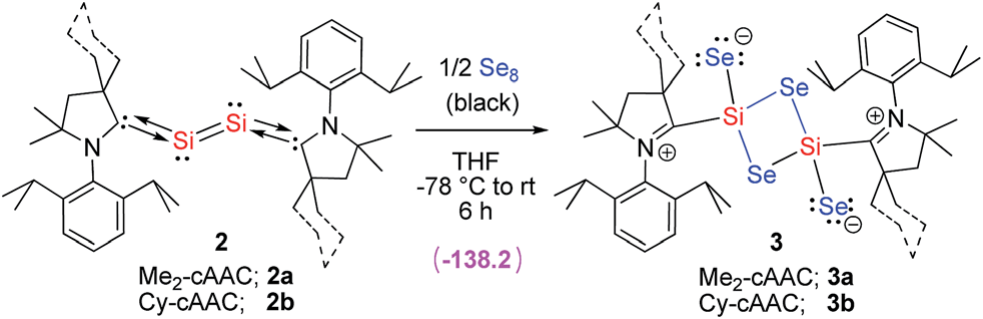
Synthesis of compound **3** from **2**. The energy term in parenthesis is Δ*G* (kcal mol^–1^) at M06-2X/TZVP/SMD//M06-2X/SVP level of theory for **2a** to **3a**.

After the first synthesis report of N-heterocyclic carbenes (NHCs) in 1991, they have been utilized as strong σ-donating ligands in different areas of chemistry.^8^ The mono-atomic and diatomic variants of silicon are stabilized by NHCs and cAACs in form of L: → Si(0) = Si(0) ← :L, and L: → Si(0) ← :L [L: = NHC or cAAC (cyclic alkyl(amino)carbene)].^9,10^ Several variants of phosphorus are stabilized by NHCs and cAACs, respectively.^10,11^ G. Bertrand *et al.* have stated^10^ that carbene stabilized variants of these elements are synthesized not only for academic curiosity but also for their solubility in organic solvents. Since organic ligand-anchored variants are more soluble in organic solvents, their chemical transformations are much easier and faster. They are suggested to be utilized for the synthesis of the smaller units of the larger framework/polymer such as silicon dioxide (Si_2_O_4_).^10^ Very recently, the syntheses and characterizations of (NHC)_2_Si_2_O_3_ and (NHC)_2_Si_2_O_4_ were reported by Robinson *et al.* ^12*a*^ Reaction of (NHC)_2_Si_2_ with excess of oxygen donor (N_2_O or O_2_) led to the isolation of free NHC and uncharacterized decomposed product of silicon oxide. The light yellow colored (NHC)_2_Si_2_O_3_ was isolated only when three equivalents of N_2_O are allowed to react with (NHC)_2_Si_2_. Note, that (NHC)_2_Si_2_O_3_ does not convert to higher oxide analogue (NHC)_2_Si_2_O_4_ when the former compound is further reacted with N_2_O. The synthesis of colorless compound (NHC)_2_Si_2_O_4_ is successful only when (NHC)_2_Si_2_ is reacted with two equivalents of molecular oxygen (O_2_).^12*a*^ Y. Apeloig has summarized the compounds containing Si=O double bonds giving high emphases on NHC stabilized Si_2_O_3_ and Si_2_O_4_ oxides.^12*b*^ However, monomeric or dimeric variants of SiSe_2_ (**B**, **C**; Scheme 1) in the molecular form are even not reported by low temperature matrix-isolation. Herein, we report on the synthesis and characterization of carbene-stabilized (SiSe_2_)_2_ in the molecular form of (cAAC)_2_Si_2_Se_4_ (**3**). The reaction employed cAAC-supported diatomic silicon(0) (cAAC)_2_Si_2_ (**2**) which is reacted with black selenium powder in the temperature range of –78 °C to rt (Scheme 2). Moreover, the stability and bonding of **3** are studied by theoretical calculations.

## Result and discussion

Both cAAC and NHC form stable adducts with SiCl_4_.^9a,c^ The colorless adducts (cAAC) → SiCl_4_ (**1a**, Me_2_-cAAC; **1b**, Cy-cAAC) are obtained when cAACs react with SiCl_4_ in 1 : 1 molar ratio in THF.^15a,9c^ The diatomic silicon(0) (cAAC)_2_Si_2_ (**2a–b**) has been synthesized by complete dechlorination of **1** with four equiv of KC_8_ in THF (see ESI†). **2a–b** are isolated as dark black-purple needles from *n*-hexane solution (see ESI for details, Schemes S1–S2†). The yield of **2a** is 52% which is higher than that of red colored (NHC)_2_Si_2_ (21%)^9*a*^ but comparable to that of **2b**.^9*c*^ Theoretical calculation showed that cAAC is more firmly bound to the Si_2_ unit and thus **2** is even characterized by EI-mass spectrometry,^9*c*^ while its NHC analogue (NHC)_2_Si_2_ is not. The latter species dissociates under such conditions and only free NHC is found in the mass spectrum. This is due to the weaker π-accepting property^13^ which makes NHC-containing compounds more labile.^14^

(NHC)_2_Si_2_,^9*a*^ and (cAAC)_2_Si_2_ (**2a**, Me_2_-cAAC; **2b**, Cy-cAAC) are studied by solid state ^29^Si NMR at room temperature (rt) to compare the electronic environments of the silicon atoms. (NHC)_2_Si_2_ shows its ^29^Si resonance at 202.4–204.3 ppm which is close to the corresponding value of 224.5 ppm (singlet) in solution.^9*a*^ Analogous measurement on **2a** exhibits a singlet at 254.6 ppm, while two resonances (190.1 and 318.3 ppm) are observed for **2b**. Note, that the calculated [(190.1 + 318.3)/2 = 254.2] mean value is ∼254 ppm. There is no resonance around 254 ppm in the solid state ^29^Si NMR of **2b** suggesting that the two silicon atoms are not equivalent. The changes in the chemical shift values of NHC and cAAC derivatives are attributed to the higher π-accepting property of cAAC over NHC.^16^ However, the reason behind the two well separated (128.2 ppm) ^29^Si resonances in **2b** is not apparently understood. The temperature dependent X-ray single crystal studies revealed that both **2a** and **2b** crystallize in the triclinic *P*1 space group. The center of inversion in between the two silicon atoms in **2b** is absent at 100 K (see ESI†), but it is present at 23 K.^9*c*^ In comparison, the center of inversion in between the two silicon atoms in **2a** is present even at 100 K (see ESI†). Structural comparison between **2a** and **2b** clearly shows that the carbene moieties are evenly disordered in **2a** but not in **2b** (Fig. S5–S8†). The combined solid state NMR and temperature dependent X-ray structural correlation unambiguously clarify the acute differences of electronic environments of silicon atoms in **2a–b**. In the solution phase both **2a** and **2b** have a similar coordination environment and hence their ^29^Si resonances are close to each other (252.3 ppm for **2a** and 249.1 ppm for **2b**) (Schemes S5–S6, Fig. S12–S13, see ESI† for details).

Compound **2** is dissolved in THF to obtain a dark purple solution which is cooled to –78 °C and passed into another flask containing black selenium powder (**2** : Se = 1 : 4 molar ratio). A light green color is obtained after stirring the solution for ten minutes. The green color of the solution is becoming more intense when stirring is continued for twenty minutes. Then the mixture is stirred for 3 h to obtain a brown solution with unreacted selenium powder. Stirring is continued for another 2.5 h to produce a clear orange solution. The volume of the THF solution is reduced to 2 mL under vacuum. Finally, 3 mL of toluene are added and the orange solution is stored at –32 °C in a freezer to form small orange blocks/needles of **3** in 30–32% yield. Compound **3b** is comparatively more soluble in THF than **3a**. Very recently the synthesis and isolation of (NHC)_2_Si_2_O_3_ and (NHC)_2_Si_2_O_4_ were reported by reacting (NHC)_2_Si with N_2_O and O_2_, respectively.^12^ The formation of (NHC)_2_Si_2_O_3_ and (NHC)_2_Si_2_O_4_ depends on the source of oxygen. Importantly, when (NHC)_2_Si_2_O_3_ was further reacted with N_2_O led to the isolation of decomposed by-products and thus (NHC)_2_Si_2_O_4_ was not obtained. It is well known that NHC mostly favors the formation of coordinate σ-bonds while cAAC forms both coordinate and electron sharing covalent σ-bonds depending on the electronic situation of the involved silicon atom.^15^

We have carried out the reaction of **2a** and Se-powder in 1 : 2, 1 : 3, and 1 : 4 molar ratios. The reaction in a 1 : 2 molar ratio does not produce the green intermediate color. A dark brown solution was obtained. The solvent was removed and the dry residue was extracted with *n*-hexane to obtain a brown filtrate and crystalline **3a** in 10–12% yield. The concentrated *n*-hexane solution was stored at –32 °C in a freezer. No crystals were formed. The removal of solvent (*n*-hexane) produced an oily material. The reaction in a 1 : 3 molar ratio proceeds first to a lighter green intermediate color. In the following step a lighter orange solution was obtained from which the crystalline powder of **3a** was isolated in 17–20% yield.

**Scheme 3:**
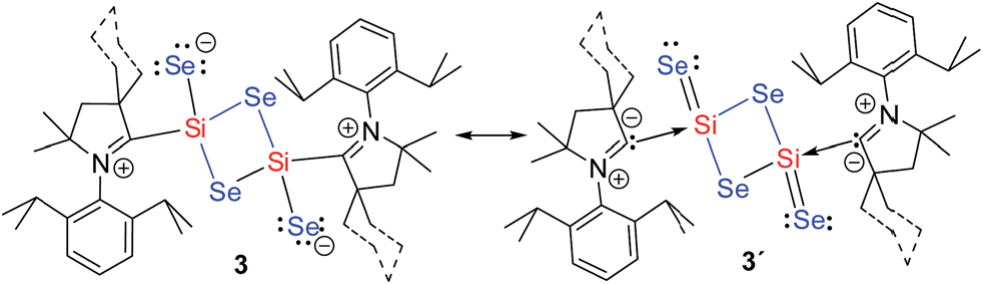
Resonance structures of compound **3**.

The crystals of **3a–b** are stable in air for several days and retain their dark orange color for a week while THF solutions of **3a–b** slowly loose their color when exposed to air. Orange powders of **3a–b** decompose above 285 °C (**3a**), 245 °C (**3b**) to give light yellow solids of cAAC=Se.^17^ This is concluded from mass spectrometry (see ESI†). Compound **3a** was further characterized by EI-MS mass spectrometry (*m*/*z* (100%); 944.2) (see ESI†). The UV-vis spectra of compounds **3a–b** recorded in THF solution show absorption bands at 422 nm (**3a**) and 402 nm (**3b**), respectively (see ESI†) which are close to the values obtained from theoretical TD-DFT calculation (400–440 nm; Tables S10 and S11†). Relative transitions are explained from KS-MO of **3a** shown in Fig. S15.† The infrared (IR) spectrum of **3a** (measured in the range of 400–4000 cm^–1^) showed a sharp absorption band at 547 cm^–1^. It is close to the theoretically calculated values of 533.3 cm^–1^ (*ν*_Si=Se_) and 355.1 cm^–1^ (*ν*_Si–Se_) of **3a**. Additionally, **3a** is investigated by Raman spectroscopy (see ESI†). Raman spectra are recorded on solid sample of **3a** which exhibit Raman bands at 1490.9 cm^–1^ and 1475.9 cm^–1^ with a shoulder. A strong Raman band (*ν*_Si=Si_)^9*c*^ was observed at 478 cm^–1^ for (cAAC)_2_Si_2_ which is not present in **3a**. Both the compounds **3a–b** are studied by solution and solid state NMR measurements. ^1^H and ^13^C NMR resonances are very broad and hence not much informative (Fig. S11†). ^29^Si and ^77^Se NMR resonances are not observed. The zwitterionic nature (Schemes 2 and 3) of compound **3** might be the reason for the broadening of the NMR resonances. However, the corresponding chemical shift values of carbene carbon, silicon, and selenium atoms are theoretically calculated and given in the ESI.†

**Fig. 1:**
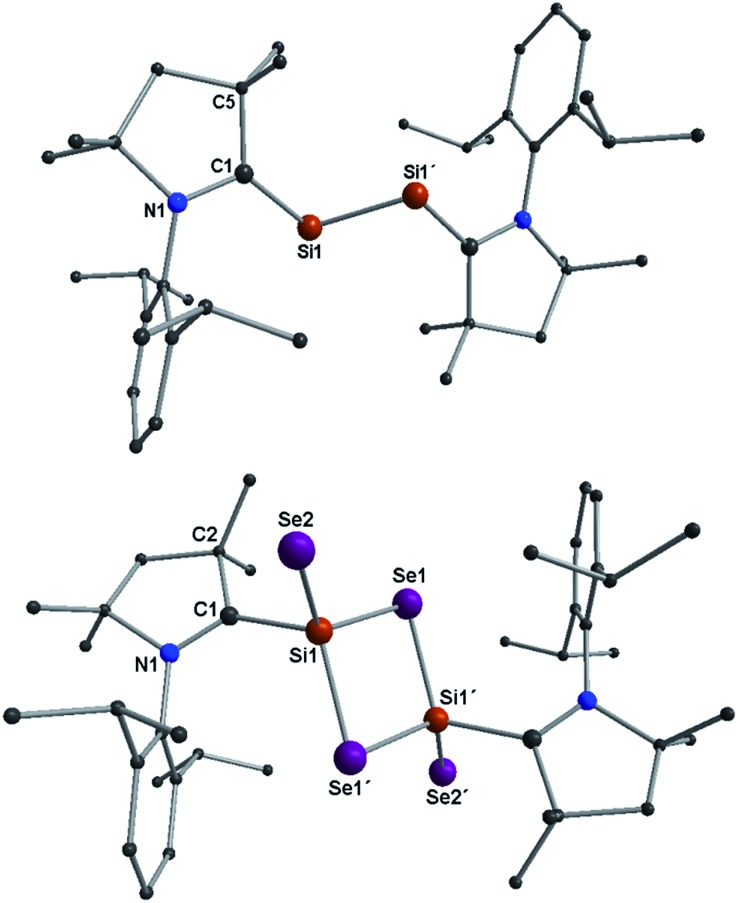
Molecular structures of compounds **2a** (top), **3a** (bottom). H atoms are omitted for clarity. Selected experimental [calculated at R-M06-2X/SVP for the singlet state] bond lengths [Å] and angles [°] (as averages of two independent molecules): for **2a**/**3a**; C1–Si1A 1.887(4)/1.931(4) [1.877/1.947], Si1A–Si1A′ 2.254(2)/3.056 [2.244/3.083], C1–N1 1.342(5)/1.311(5) [1.343/1.309]; C1–Si1A–Si1A′ 103.67(14)/123.94 [102.16/123.51]. For **3a**; Si1–Se1 2.2874(10) [2.318], Si1–Se1′ 2.3046(10) [2.319], Si1–Se2 2.1510(10) [2.156]; Se1–Si1–Se1′ 96.55(4) [96.52], Si1–Se1–Si1′ 83.45(4) [83.32], C1–Si1–Se2 97.86(11) [96.58], C1–Si1–Se1 109.85(11) [107.85], C1–Si1–Se1′ 113.81(11) [113.33], Se2–Si1–Se1 119.05(4) [121.1], Se2–Si1–Se1′ 120.35(4) [121.67].

Structural descriptions of **2a–b** are given in ESI.† Compound **3a** crystallizes in the space group *P*2_1_/*n* and possesses a center of inversion within the molecule. The asymmetric unit of **3a** contains the (Me_2_-cAAC)SiSe_2_ fragment. The complete molecular structure of **3a** generated from applying inversion symmetry is shown in Fig. 1.

The Se=Si(*μ*-Se)_2_Si=Se unit (alike **C** shown in Scheme 1) is coordinated by two Me_2_-cAAC ligands. Both silicon atoms adopt a four coordinate distorted tetrahedral geometry. The C_cAAC_–Si bond length of **3a** is 1.931 (4) Å which is close to that in **1a** (1.944 (2) Å)^15*a*^ but larger than that in **2a** (1.887(4) Å) (Table S1†). This suggests that the bond between carbene carbon and silicon is a donor bond (C_cAAC_ → Si) (Scheme 3), rather than a donor–acceptor partial double bond^9*c*^ in **2a** as illustrated in Scheme 2. The Si1–Se1 and Si1–Se2 bond distances of **3a** are 2.2874(10)/2.3046(10), and 2.1510(10) Å, respectively, suggesting single bond (Si1–Se1, Si1–Se1′) and double bond (Si1=Se2) character (Scheme 3).^14^ The Si1–Se1/Se1′ bond distances are close to the values reported for (SiSe_2_)_*n*_ (**A**) (2.275 Å). The Se–Si–Se bond angle in Si_2_Se_2_ four membered ring of **3a** is 96.55(4)° which is sharper (by ∼3.5°) than that of **A** (100.0(1)°).^3*b*^ This might be due to the coordination of Me_2_-cAAC to each silicon atom. The Si_2_Se_4_ core of compound **3a** is structurally similar to that of (NHC)_2_Si_2_O_4_ compound reported by Robinson *et al.* ^12*a*^ The silicon–silicon bond distance is 3.056 Å in **3a** while that of (NHC)_2_Si_2_O_4_ is 2.3980(11) Å which is due to the larger size of the selenium atoms.

We have performed DFT calculations to illustrate the electronic structure and bonding scenario of **3a** (refer Computational Details in ESI†). The optimized geometry of **3a** at the M06-2X/SVP level shows a strong resemblance with the X-ray crystal structure of **3a** (Fig. 1 and S14†). The electronic structure and bonding features of **3a** are illustrated using NBO analysis as implemented in Gaussian09. The calculations reveal that C1 is connected to Si1 by a single bond with electron occupancy of 1.94701 e which is primarily located on the C1 (77%) center. The N1–C1 bond in **3a** is significantly shorter (1.309 Å) than in **2a** (1.343 Å) due to the strong π-bonding interaction to disrupt C1←Si1 back donation. This finding also reveals that the C1 is bound to the Si1 as a singlet carbene donor (C1→Si). On the other hand Si1 also binds to Se2 by a single bond and Se contains three lone pairs. But the Si1–Se2 bond length is 2.15 Å which is significantly shorter than the single bond length (2.28 Å and 2.07 Å in H_3_Si–SeH and H_2_Si=Se, respectively). It is surprising to see that the lone pair occupancies on the Se2 are 1.960, 1.713, and 1.717 e, respectively. The lowering in occupancy of the last two lone pairs can be envisaged as some sort of donor–acceptor type interaction with the Si1 atom, in turn making the bond shorter (Scheme 3).

Topological and topographical analyses are also performed for further illustration of the bonding features in **3a** using QTAIM (Quantum Theory of Atoms in Molecules) calculations (see computational details in ESI†).

**Fig. 2:**
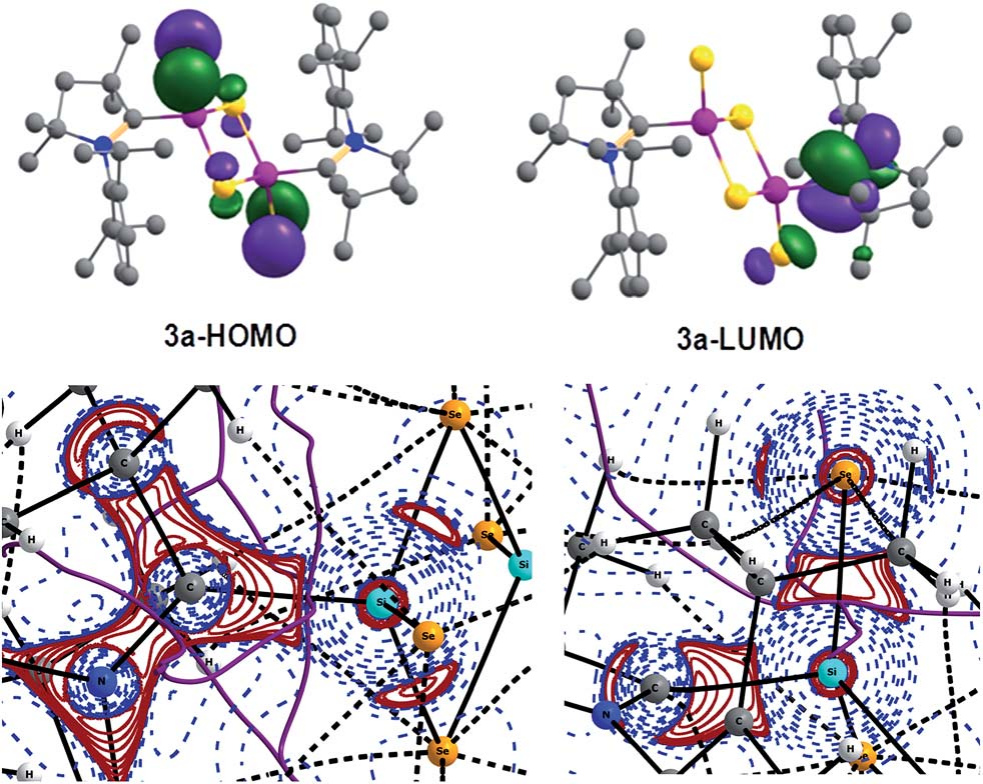
(Top) Computed KS-MO of **3a** at R-M06-2X/TZVP//R-M06-2X/SVP level. Hydrogen atoms are omitted for clarity. (bottom) Laplacian distribution [∇^2^ *ρ*(*r*)] in N1–C1–Si1 (left) and C1–Si1–Se2 plane (right) of **3a**. Solid lines indicate the areas of charge concentration (∇^2^ *ρ*(*r*) < 0) while dotted lines mean the charge depletion (∇^2^ *ρ*(*r*) > 0). The range of contours of the Laplacian is –8 × 102 to +8 × 102. Solid lines connecting atomic nuclei (black) are the bond paths and those lines (purple) separating the atomic basins indicates the zero-flux surface crossing the molecular plane.

The electron density, *ρ*(*r*), at the (3,–1) bond critical points (BCPs) of C1–Si1 (0.095) and Si1–Se2 (0.104) bonds along with the respective Laplacian [∇^2^ *ρ*(*r*); +0.240 and +0.051] indicate closed-shell interaction *i.e.*, donor–acceptor bond (Table S9†). This is further supported by 2D Laplacian plot of (3,–3) critical points (Fig. 2, bottom). The Delocalization Index (DI) value of C1–Si1 (0.43) is lower than for an ordinary C–Si bond in H_3_C–SiH_3_ (0.55), indicating the presence of weak C1–Si1 donor type bonding. In case of the Si1–Se2 bond the DI value donor (0.6; Si1→Se2) is close to that of the Si–Se single bond (0.58) in H_3_Si–SeH in accordance with the NBO results discussed above. The real bonding in **3** is a combination of two resonating structures as shown in Scheme 3. In contrast the lower but positive value of Laplacian (+0.051) indicates closed-shell binding nature to lesser extent. We presume that the more electronegative Se is reluctant to share its lone pairs to the adjacent Si center in turn contributing towards equal sharing between the partners.

## Conclusions

**Fig. 3:**
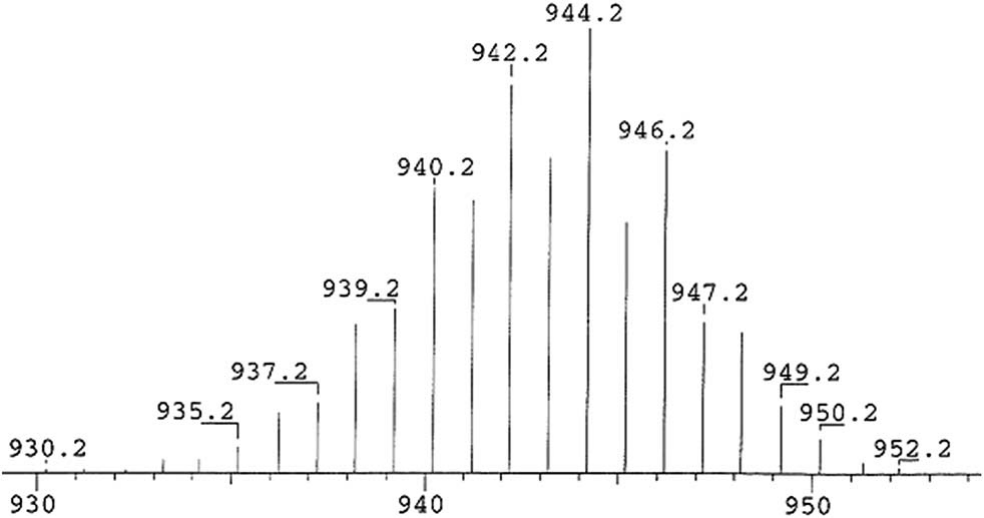
(a) Experimental EI-mass spectrum of compound **3a**.

We have shown that carbene coordinated diatomic silicon(0) species (cAAC)_2_Si_2_ (**2a–b**) can react with black selenium powder in a 1 : 4 molar ratio in THF at –78 °C to rt to produce molecular compound (cAAC)_2_Si_2_Se_4_ (**3a–b**). Orange powders of **3a–b** are stable under an inert atmosphere for nearly a month and decompose above 245 °C. Dark orange crystals of **3a–b** retain their colors in air for a week. They are soluble in polar organic solvents, such as THF, while partially soluble in toluene and *n*-hexane. The molecular structure of **3a** was confirmed by X-ray single crystal diffraction and EI-mass spectrometry (Fig. 3). A comparison between the bond parameters of **2a** and **3a** unambiguously led to the conclusion that the Si_2_(0) unit in **2a** is stabilized by a donor acceptor type bond between 2cAAC and (0)Si=Si(0), while its derivative Si_2_Se_4_ of **3a** has been prevented from undergoing polymerization by strong σ-donation of the cAAC molecules (Schemes 2 and 3). The bonding and stability of **3a** have been further studied by theoretical calculations. To the best of our knowledge, this is the first report on a neutral ligand-stabilized molecular Si_2_Se_4_ species.^18,19^
