# Supplementary material for: A soluble molecular variant of the semiconducting silicondiselenide
Source: Chem Sci. 2015 Jun 18;6(9):5230–4. doi: 10.1039/c5sc01516b (PMC5500843; doi:10.1039/c5sc01516b)
Supplement: Supplementary file 1 [file SC-006-C5SC01516B-s001.pdf]

Electronic Supplementary Material (ESI) for Chemical Science.
This journal is © The Royal Society of Chemistry 2015

# A Soluble Molecular Variant of the Semiconducting Siliconediselenide

Kartik Chandra Mondal, Sudipta Roy, Birger Dittrich,* Bholanath Maity, Sayan Dutta, Debasis Koley,* Suresh Kumar Vasa, Rasmus Linser, Sebastian Dechert, and Herbert W. Roesky,*

## Supporting Information

**Content:**

(S1) Syntheses of compounds **1**, **2**, and **3**
(S2) UV-visible spectroscopy
(S3) Crystal data of **2**, **3**
(S4) Solid state NMR
(S5) Theoretical calculation
(S6) Raman spectra of **3a**
(S7) NMR spectra
(S8) EI-mass spectra
(S9) References

### (S1) Synthesis

All reactions and handling of reagents were performed under an atmosphere of dry nitrogen or argon using standard Schlenk techniques or a glove box where the $O_2$ and $H_2O$ levels were usually kept below 1 ppm. $Me_2$-cAAC, Cy-cAAC, ($Me_2$-cAAC)→$SiCl_4$ (**1a**) and (Cy-cAAC)→$SiCl_4$ (**1b**) were prepared according to literature methods.[S1-S3] Solvents were purified with the M-Braun solvent drying system. Solution NMR spectra were recorded on Bruker Avance 200, Bruker Avance 300, and Bruker Avance 500 MHz NMR spectrometers. Deuterated NMR solvent THF-$d_8$ and $C_6D_6$ were dried by stirring for 2 days over Na/K alloy followed by distillation in vacuum. EI-MS spectra were obtained with a Finnigan MAT 8230 or a Varian MAT CH5 instrument (70 eV) by EI-MS methods. Elemental analyses were performed by the Analytisches Labor des Instituts für Anorganische Chemie der Universität Göttingen. Melting points were measured in sealed glass tubes on a Büchi B-540 melting point apparatus.

$(Me_2\text{-cAAC})_2Si_2$ (**2a**): The mixture of $(Me_2\text{-cAAC})\rightarrow SiCl_4$ (**1**) (779 mg; 1.71 mmol) and $KC_8$ (924 mg; 6.84 mmol) was taken into a 100 mL round bottom flask and 40 mL of THF was added at -78 °C. The mixture was stirred for 1.5 h and filtered through celite to get a dark red violet filtrate which was dried under vacuum and extracted with *n*-hexane (60 mL). The evaporation of solvent under vacuum gave dark black needles from the violet solution in 52% yield. The X-ray single crystal analysis revealed the molecular structure of **2a**. Elemental analysis found in % (calcd) for $C_{40}H_{62}N_2Si_2$: C, 76.30 (76.61); H, 9.89 (9.96); N, 4.38 (4.46). Melting point 180-181 °C, decomposition point 200-205 °C, Uv-visible bands at 516.7, 397.0 , and 377.8 nm. The needles of **2a** are sensitive to air and moisture. They loose their colors from dark purple to colorless powder of $Me_2$-cAAC=O[S5] within ten min when exposed to air.

**$^1$H NMR** (500 MHz, 298 K, $C_6D_6$, ppm) $\delta$: 7.12 (d, $J$ = 3.3 Hz, 1H), 7.10 (d, $J$ = 8.2 Hz, 4H), 7.08 (d, $J$ = 2.6 Hz, 1H), 3.12 (dq, $J$ = 13.3, 6.6 Hz, 4H), 1.77 (s, 4H), 1.71 (s, 12H), 1.69 (d, $J$ = 6.6 Hz, 12H), 1.21 (d, $J$ = 6.7 Hz, 12H), 1.06 (s, 12H).

**$^{13}$C NMR** (126 MHz, 298 K, $C_6D_6$, ppm) $\delta$: 236.69 ($C_{carbene}$), 148.55, 135.24, 129.34, 125.67, 71.59, 54.74, 50.29, 32.18, 29.85, 29.45, 28.82, 25.17.

**$^{29}$Si NMR** (99 MHz, 298 K, $C_6D_6$, ppm) $\delta$: 252.34.

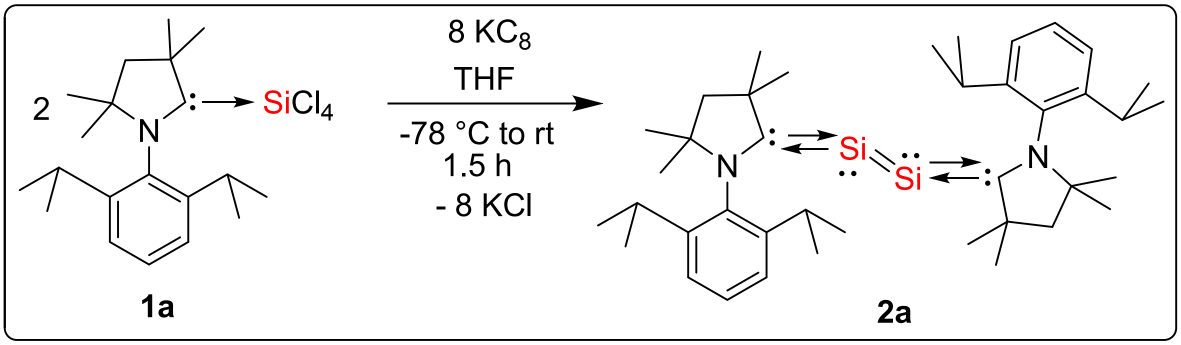


**Scheme S1**: Synthesis of compound **2a** from **1a** (cAAC = $Me_2$-cAAC).

Compound **2b**: The mixture of $(Cy\text{-cAAC})\rightarrow SiCl_4$ (**1b**) (1680 mg; 3.40 mmol) and $KC_8$ (924 mg; 13.68 mmol) was taken into a 100 mL round bottom flask and 50 mL of THF were added at -78 °C. The mixture was stirred for 1.5 h and filtered through celite to get a dark red violet filtrate which was dried under vacuum and extracted with *n*-hexane (2×70 mL). The evaporation of solvent under vacuum gave dark black needle like crystals of **2b** from the purple solution in 54% yield. Elemental analysis found in % (calcd) for $C_{46}H_{70}N_2Si_2$: C, 78.18 (78.12); H, 9.86 (9.97); N, 3.95 (3.96). Melting point 188-190 °C, Uv-vis bands at 528 and 416 nm.

**$^1$H NMR** (500 MHz, 298 K, $C_6D_6$, ppm) $\delta$: 7.13 (t, $J$ = 4.9 Hz, 3H), 7.11–7.09 (m, 3H), 3.16–3.06 (m, 4H), 2.97 (dd, $J$ = 18.0, 7.5 Hz, 4H), 1.84 (s, 4H), 1.69 (d, $J$ = 6.6 Hz, 12H), 1.63–1.56 (m, 10H), 1.28 (dd, $J$ = 15.2, 7.8 Hz, 6H), 1.23 (d, $J$ = 6.7 Hz, 12H), 1.09 (s, 12H).

**$^{13}$C NMR** (126 MHz, 298 K, $C_6D_6$, ppm) $\delta$: 234.77 ($C_{carbene}$ ), 148.04, 134.99, 128.79, 125.27, 71.53, 55.30, 48.63, 38.66, 29.81, 29.05, 28.26, 25.84, 24.92, 23.83.

**$^{29}$Si NMR** (99 MHz, 298 K, $C_6D_6$, ppm) $\delta$: 249.13.

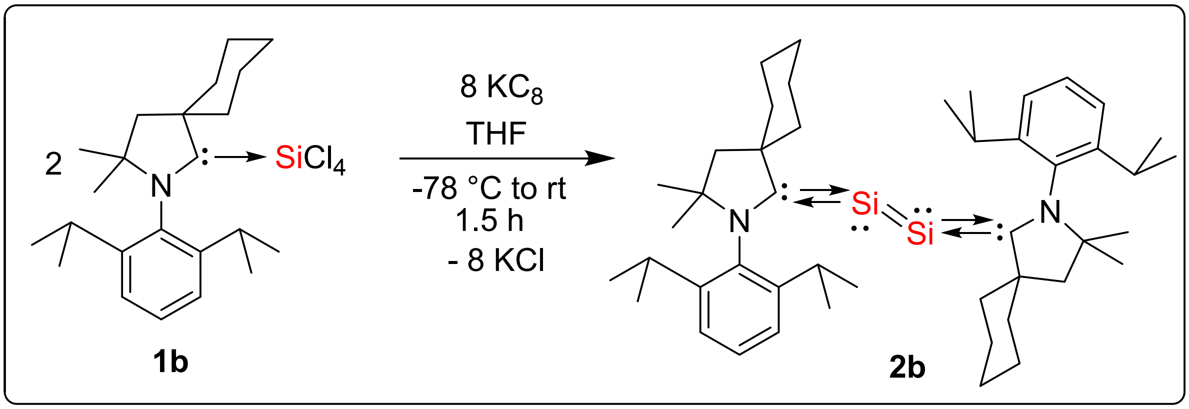


**Scheme S2**: Synthesis of compound **2b** from **1b** (cAAC = Cy-cAAC).

Compound **3a**: Compound **2a** (0.2 mmol; 126 mg) is dissolved in THF (25 mL) to obtain a dark purple solution which is cooled to -78 °C and passed into another flask containing black selenium powder (0.8 mmol; 64 mg) (**2a**:Se = 1:4 molar ratio). The temperature of the solution is raised to room temperature over 30 min. The mixture is stirred for 3 h to obtain a brown solution with unreacted selenium powder. Stirring was continued for another 2.5 h to produce a clear orange solution. The volume of THF solution is reduced to 2 mL under vacuum. Finally, 3 mL of toluene are added and the orange solution is stored at -32 °C in a freezer to form small orange blocks of **3a** in 30% yield. Elemental Analysis for $C_{40}H_{62}N_2Se_4Si_2$ (cald.): C, 50.86 (50.95); H, 6.71 (6.63); N, 2.93 (2.97). UV-vis absorption band at 422 nm (recorded on solid sample). The infrared (IR) spectrum of **3a** (measured in the range of 400-4000 $cm^{-1}$) showed a sharp absorption at 547 $cm^{-1}$. Additionally, **3a** is studied by Raman spectroscopy (see SI). Orange powders of **3a** decompose above 285 °C to give light yellow solids of $Me_2$-cAAC=Se.[S4] **3a** is partially soluble in both *n*-hexane and THF. EI-MS mass spectrum of **3a**: (*m/z*(100%); 944.2).

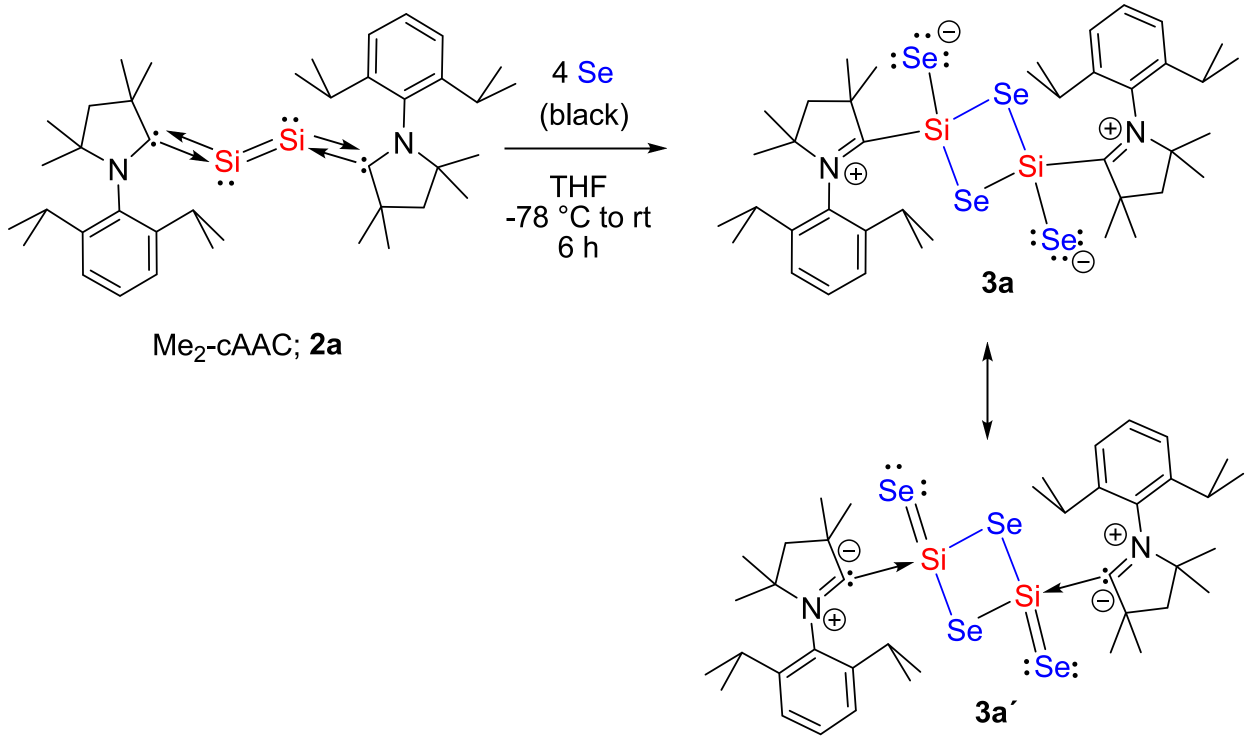


**Scheme S3**: Synthesis of compound **3a** from **2a**.

Compound **3b**: Compound **2b** (0.2 mmol; 141 mg) is dissolved in THF (25 mL) to obtain a dark purple solution which is cooled to -78 °C and passed into another flask containing black selenium powder (0.8 mmol; 64 mg) (**2b**:Se = 1:4 molar ratio). The temperature of the solution is raised to room temperature (rt) over 30 min. The mixture is stirred for 1.5 h at rt to obtain a brown solution with unreacted selenium powder. The reaction of **2b** is faster than that of **2a**. Stirring was continued for another 2 h to produce a clear orange solution. The volume of THF solution is reduced to 2 mL under vacuum. Concentrated orange colored THF solution is stored at -32 °C in a freezer to form small orange rods of **3b** in 32% yield. Compound **3b**: Elemental Analysis for $C_{46}H_{70}N_2Se_4Si_2$ (cald.): C, 53.8 (54.0); H, 6.86, (6.90); N, 2.71 (2.74). **3b** is partially soluble in *n*-hexane but completely soluble in THF. Orange powders of **3b** decompose above 245 °C.

The UV-vis absorption band of **3b** (recorded in THF) at 402 nm. Compound **3b** is comparatively more soluble in THF than **3a**.

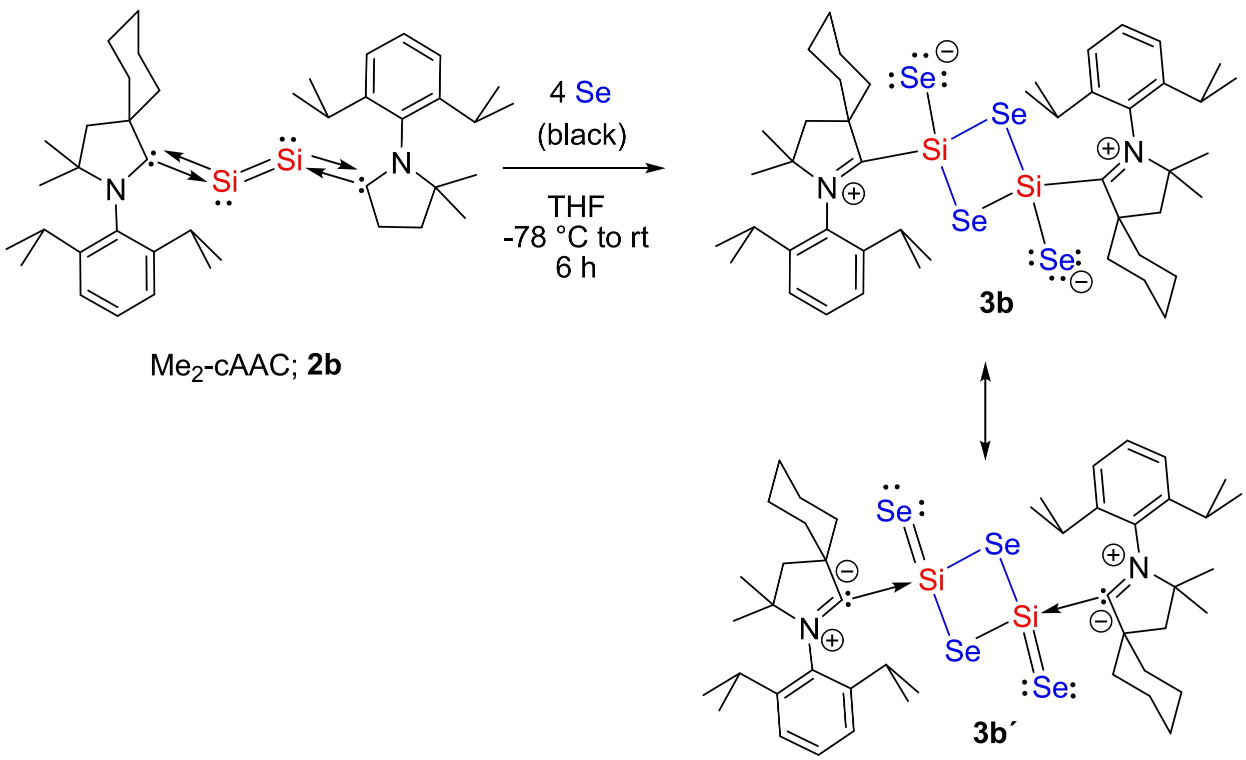


**Scheme S4**: Synthesis of compound **3b** from **2b**.

Common observations for **3a-b**: The crystals of **3a-b** are stable in air for several days and retain their dark orange color for a week while THF solutions of **3a-b** slowly loose their colors when exposed to air. The powders of **3a-b** are relatively less stable in air when compared with those of their crystals. Compounds **3a-b** have a strong smell which is common for selenium containing organic compounds. Both the compounds **3a-b** are studied by solution and solid state NMR measurements. $^{1}$H and $^{13}$C NMR resonances are very broad (both in solid state and in solution) and hence not much informative. $^{29}$Si and $^{77}$Se NMR resonances are not observed. However, the corresponding chemical shift values of carbene carbon, silicon, and selenium atoms are theoretically calculated and given in the theoretical part.

**(S2) UV-visible spectroscopy**

UV-vis spectra were recorded on Varian Cary 5000 (Varian) spectrophotometer.

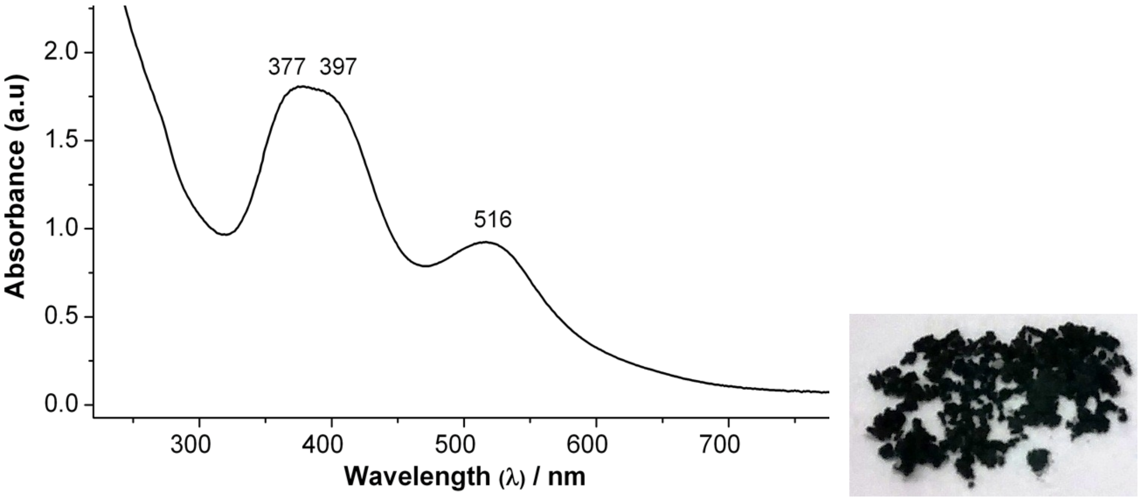


**Figure S1.** UV-visible spectrum of compound **2a** in *n*-hexane (left). Solid sample of **2a** (right). When these crystals are dissolved in organic solvents dark purple color solutions are obtained.

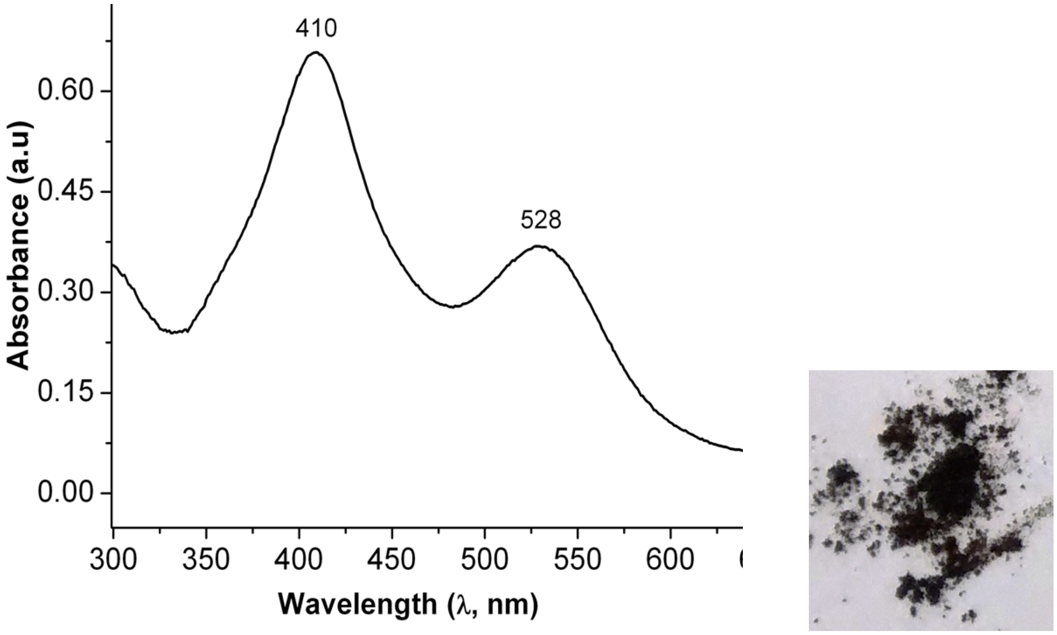


**Figure S2.** UV-visible spectrum of compound **2b** in *n*-hexane (left). Solid powdered sample of **2b** (right).

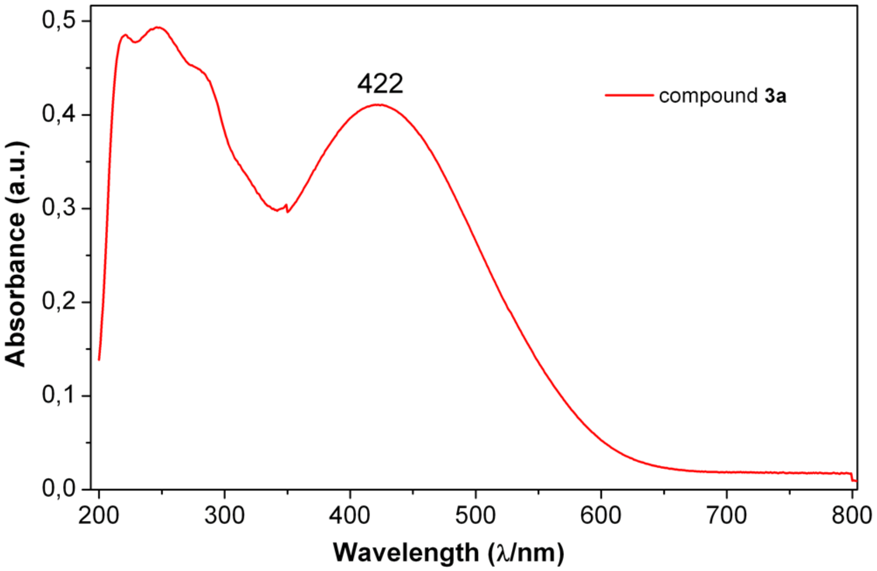

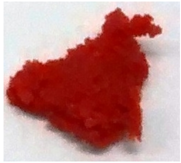

**Figure S3.** UV-visible spectrum of solid powered sample of compound **3a** (left). Solid sample of **3a** (right).

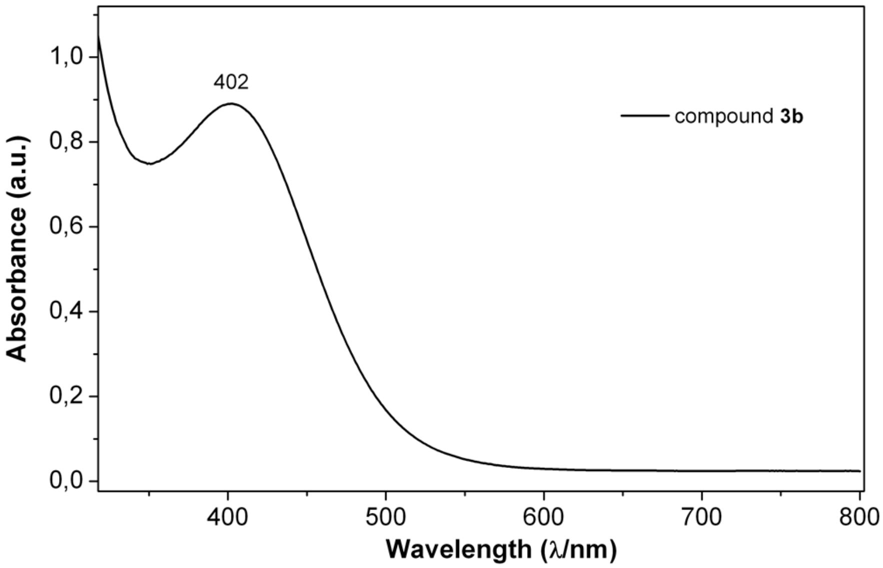

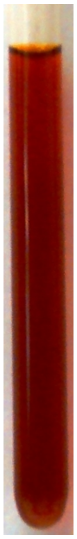

**Figure S4.** UV-visible spectrum of compound **3b** in THF (left). THF solution of **3b** (right).

## (S3) Crystal data

The molecular structures of **2a**, and **2b** were elucidated by single-crystal X-ray diffraction. Crystallographic data for **2a-b, 3a·2THF,** and $Me_2$-cAAC=O[S5] were measured on a Bruker three-circle diffractometer equipped with a SMART 6000 CCD area detector and a CuK alpha rotating anode. Integrations were performed with SAINT 8.27B. Intensity data for all three compounds were corrected for absorption and scaled with SADABS.[S6] Synchrotron data from XDS integration were converted with XDS2SAD (G. Sheldrick) prior to use with SADABS. All crystals were measured at a temperature of 100 K. Structures were solved by direct methods and those of **2a** and **2b** refined by full-matrix least-squares methods on $F^2$ with the program SHELXL12[S7] using anisotropic displacement parameters for all non-hydrogen atoms. Anomalous dispersion for the chosen wavelength was adjusted with the DISP command for the synchroton data. Unmerged intensity data and the SHELXL refinement instructions are included in the respective CIF files.

Data for $Me_2$-cAAC=O were refined with aspherical scattering factors of the invariom database.[S8] Invariom refinement led to an improvement in the crystallographic R-factor of 1.27 %. Refinement was carried out with XDLSM from the XD suite of programs.[S9]

The X-ray diffraction study on black needle shaped single crystals of compounds $(Me_2\text{-cAAC})_2Si_2$ (**2a**) and (Cy-cAAC)$_2$Si$_2$ (**2b**) show that both crystallize in the triclinic space group *P*-1. The crystal data of both **2a** and **2b** were collected at 100 K. Structural analysis of **2** shows silicon atoms are disordered. The structure analysis exhibits that only half of the molecule is contained in the asymmetric unit.

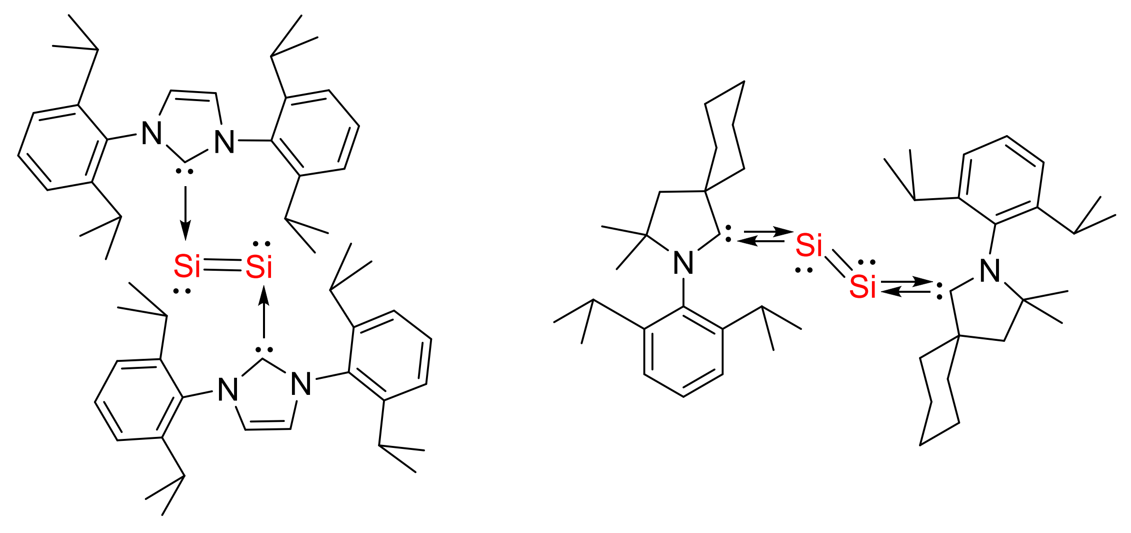


(NHC)$_2$Si$_2$ (Cy-cAAC)$_2$Si$_2$ (**2b**)

**Scheme S5**: The bond between NHC and $Si_2$ is a strong σ-donor type ($C_{NHC}$→Si) with negligible back bonding (left). The bond between cAAC and $Si_2$ in (Cy-cAAC)$_2$Si$_2$ (**2b**) is a strong σ-donor type ($C_{NHC}$→Si) with strong π-back donation from $C_{cAAC}$ to $Si_2$ ($C_{cAAC}$←Si) (right). Thus the bond between $C_{cAAC}$ and silicon atoms has partial

double bond character. The electronic structure of (Cy-cAAC)$_2$Si$_2$ (**2b**) was concluded as shown in Scheme S5. It is arranged from combined Raman spectroscopy and theoretical calculation.[S3]

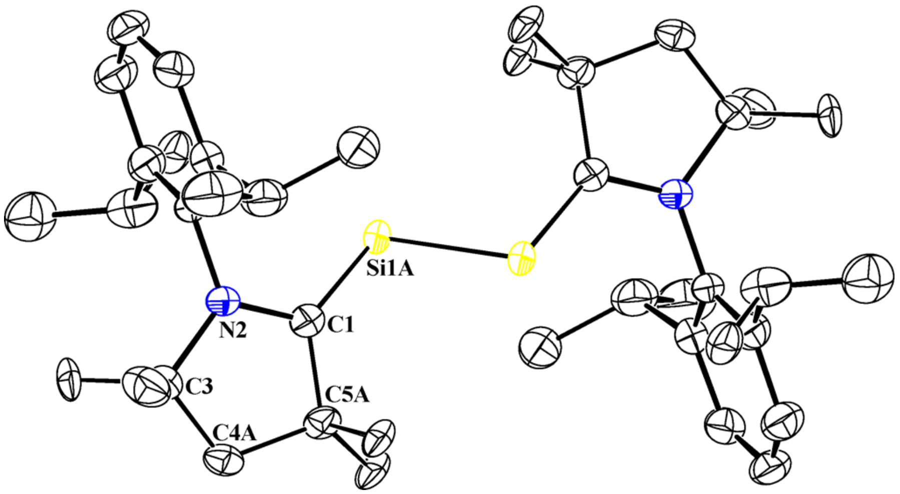


**Figure S5**. Molecular structure of **2a** (ORTEP picture with 50% probability), hydrogen atoms were omitted for clarity. (T = 100 K) (major conformer 75%)

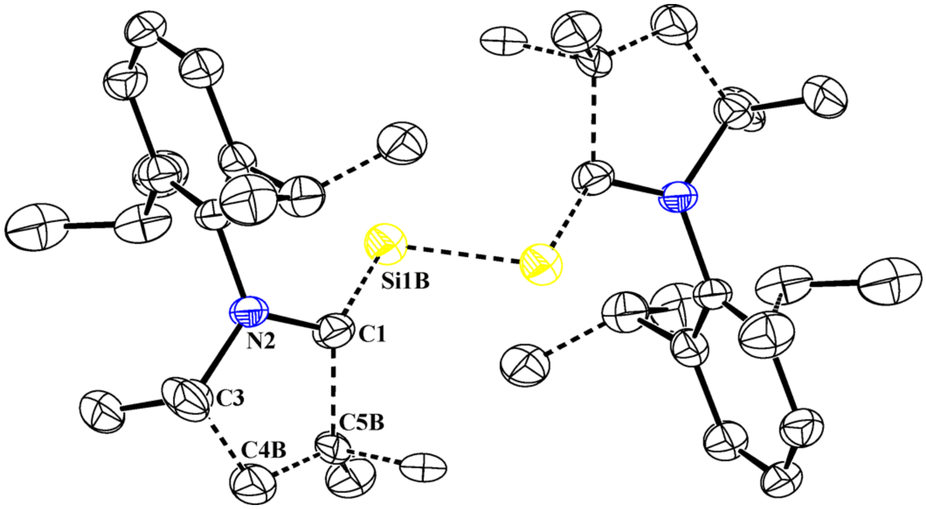


**Figure S6**. Representation of molecular structure of disordered part **2a** (ORTEP picture with 50% probability), hydrogen atoms were removed for clarity. (T = 100 K) (minor conformer 25 %). Figures S5 and S6 suggests that the Me$_2$-cAAC carbene in **2a** is evenly disordered in solid state.

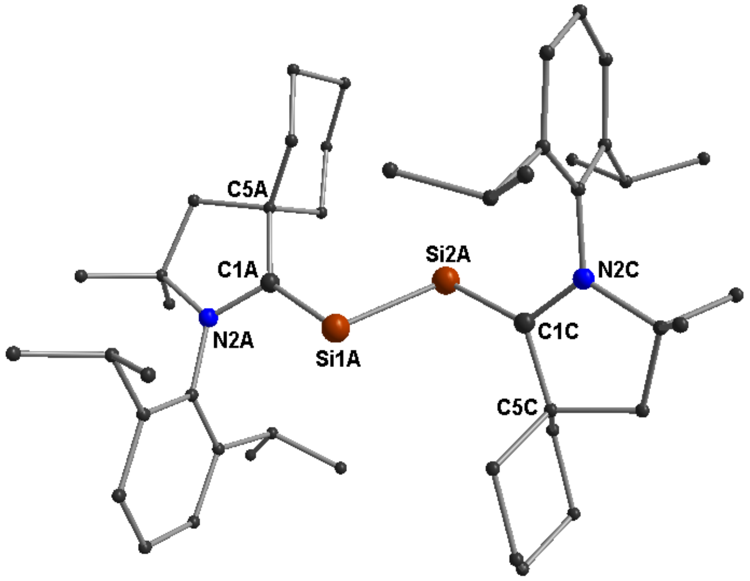


**Figure S7**. Molecular structure of (Cy-cAAC)$_2$Si$_2$ (**2b**). Hydrogen atoms were omitted for clarity. (T = 100 K)

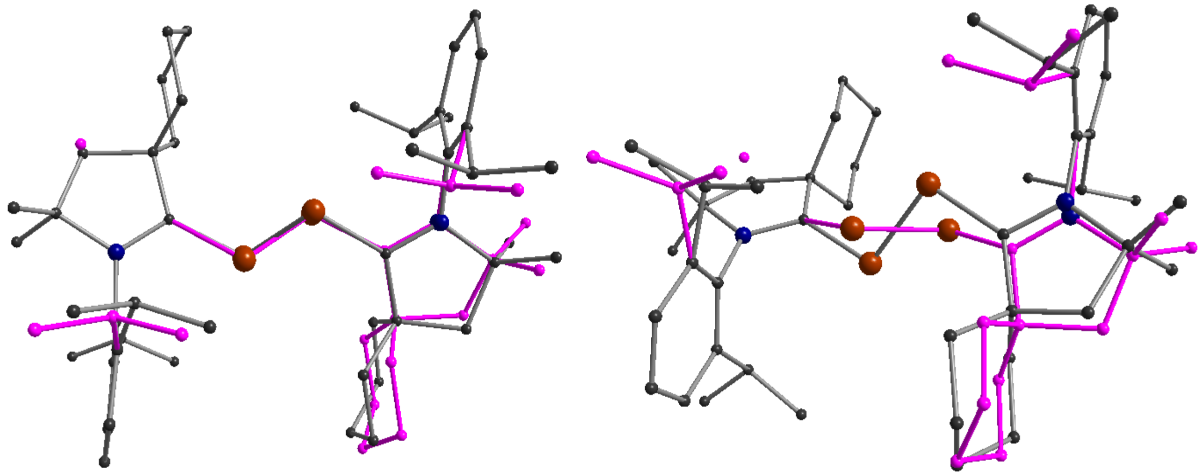

**Figure S8**. Different views on molecular structures of two conformers of **2b** (major in grey; minor in purple) in solid state. The picture at the right hand side shows that the Cy-cAAC carbene (colored in purple) is disordered. The occupancy ratio of grey:purple part is 80:20 at 100 K and ~ 97:3 at 23 K. Figures S7 and S8 suggests that the Cy-cAAC carbene in **2b** is unevenly disordered in solid state.

**Table S1.** Selected bond parameters of $(Me_2\text{-cAAC})_2Si_2$ (**2a**) and $(Me_2\text{-cAAC})_2Si_2Se_4\cdot2THF$ (**3a·2THF**).

| Compound | **2a** (at 100 K) | **2b** (at 23 K)[S3] | **2b** (at 100 K) | $(NHC)_2Si_2$ [S10] |
|---|---|---|---|---|
| Si-$C_{carbene}$ (Å) | 1.887(4) | 1.849(4)-1.876(4) | 1.836(18), 1.878(5), 1.861(4) | 1.9271(15) |
| $C_{carbene}$ -N | 1.342(5) | 1.352(3) | 1.33(2)-1.347(6) | - |
| Si-Si (Å) | 2.232(9)/2.254(2) | 2.254(3) | 2.232 (9)-2.255(4) | 2.2294(11) |
| C1-Si1-Si2/C21-Si1-Si2 (°) | 103.67(14) | 101.22 (13)/ 105.14(13) | 101.99(14), 104.24(12), 105.1(6), 97.9(3) | 93.37(5) |
| C1-Si1-Si2-C21 (°) | 180.0 | 179.16 | 179.03 | 180.0 |
| Center of inversion between two silicon atoms | yes | yes | No | yes |
| ……………………………... | .................................... | ............................. | ........................... | .......................... |
| Compound | **2a** (at 100 K) | **1a** [S2] | **3a** | |
| Si-$C_{carbene}$ (Å) | 1.887(4) | 1.944(2) | 1.931(4) | |
| $C_{carbene}$ -N | 1.342(5) | 1.303 (2) | 1.311(5) | |
| Si-Si (Å) | 2.232(9)/2.254(2) | - | 3.056 | |
| C-$C_{carbene}$ –N (°) | 105.9(19)/109.0(7) | 110.1 (4) | 110.0 (3) | |

**Table S2.** Crystal data and structure refinement of $(Me_2\text{-cAAC})_2Si_2$ (**2a**) and $(Me_2\text{-cAAC})_2Si_2Se_4\cdot2THF$ (**3a·2THF**).

| Compound | $(Me_2\text{-cAAC})_2Si_2$ ($Me_2$-cAAC = :C($CH_2$)($CMe_2$)$_2$N-2,6-$i$$Pr_2C_6H_3$ ) (**2a**) | $(Me_2\text{-cAAC})_2Si_2Se_4$ ($Me_2$-cAAC = :C($CH_2$)($CMe_2$)$_2$N-2,6-$i$$Pr_2C_6H_3$ ) (**3a·2THF**) |
|---|---|---|
| Empirical formula | $C_{40}H_{62}N_2Si_2$ | $C_{20}H_{31}NSe_2Si\cdot(C_4H_8O)$ |
| CCDC no. | 926618 | 1060365 |
| Molecular weight | 627.09 | 543.57 |
| Crystal size [mm] | 0.02 x 0.01 x 0.01 | 0.09 x 0.08 x 0.06 |
| Wavelength [Å] | 1.54178 | 1.54178 |
| Crystal system | triclinic | monoclinic, |
| Space group | *P*-1 | $P2_1/n$ |
| *a* [Å] | 9.4109(9) | 14.6952 (6) |
| *b* [Å] | 10.1841(10) | 10.0800 (4) |

| | | |
|---|---|---|
| *c* [Å] | 11.6538(12) | 17.7472 (7) |
| *α* [°] | 91.036(6) | 90.0 |
| *β* [°] | 102.694(6) | 109.567 (2) |
| *γ* [°] | 116.006(6) | 90.0 |
| *V* [Å $^3$] | 970.92(17) | 2477.03 (17) |
| *Z* | 1 | 4 |
| Temperature [K] | 100(2) | 100(2) |
| *ρ* [Mg m$^{-3}$] | 1.073 | 1.458 |
| *μ* [mm$^{-1}$] | 1.02 | 4.30 |
| *F* (000) | 344 | 1120 |
| *θ*-area [°] | 3.9 to 65.2 | 3.4 to 70.1 |
| Total number reflect. | 16983 | 22159 |
| Unique reflections | 3341 | 4611 |
| reflections with $I > 2\sigma(I)$ | 2685 | 3674 |
| $R_{int}$ | 0.0645 | 0.062 |
| Number of restraints | 130 | 264 |
| Parameters | 276 | 301 |
| *R*1 [$I$>2$\sigma(I)$] | 0.0747 | 0.0393 |
| *wR*2 [$I$>2$\sigma(I)$] | 0.1935 | 0.0922 |
| *R*1 [all data] | 0.0908 | 0.0556 |
| *wR*2 [all data] | 0.2049 | 0.1002 |
| GooF | 1.064 | 1.02 |
| Extinction coefficient | | |
| Largest diff. peak / hole max. / min. [$10^3$·e·nm$^{-3}$] | 0.452 and -0.276 | 1.01 and −0.42 |

**Table S3.** Crystal data and structure refinement of (Cy-cAAC:)$_2$Si$_2$ (**2b**) at two different temperatures (23 K[S1] and 100 K).

| Compound | (Cy-cAAC:)$_2$Si$_2$ (Cy-cAAC: = :C(CH$_2$)(CMe$_2$)(C$_6$H$_{10}$)N-2,6-*i*Pr$_2$C$_6$H$_3$ ) (**2b**) at T = 23 K[S3] | 2 (Cy-cAAC:)$_2$Si$_2$ (Cy-cAAC: = :C(CH$_2$)(CMe$_2$)(C$_6$H$_{10}$)N-2,6-*i*Pr$_2$C$_6$H$_3$ ) (**2b**) at T =100 K |
|---|---|---|
| Empirical formula | $C_{46}H_{70}N_2Si_2$ | $C_{92}H_{140}N_4Si_4$ |
| CCDC no. | 983863 | 948799 |
| Molecular weight | 707.22 | 1414.43 |
| Crystal size [mm] | 0.07 × 0.03 × 0.02 | 0.04 × 0.01 × 0.01 |
| Wavelength [Å] | 0.4769 | 0.6358 |
| Crystal system | triclinic | triclinic |
| Space group | *P*-1 | *P*-1 |
| *a* [Å] | 12.79(2) | 12.80(3) |
| *b* [Å] | 13.783(9) | 13.86(4) |
| *c* [Å] | 14.433(19) | 14.36(2) |
| *α* [°] | 66.35(6) | 66.45(7) |
| *β* [°] | 79.58(10) | 79.90(4) |
| *γ* [°] | 67.48(7) | 67.84(9) |

| | | |
|---|---|---|
| $V$ [nm$^3$] | 2152(5) | 2162(8) |
| $Z$ | 2 | 2 |
| Temperature [K] | 23 | 100 |
| $\rho$ [Mg m$^{-3}$] | 1.091 | 1.086 |
| $\mu$ [mm$^{-1}$] | 0.06 | 0.09 |
| $F$ (000) | 776 | 776 |
| $\theta$-area [°] | 1.0 to 16.9 | 2.0 to 22.6 |
| Total number reflect. | 45604 | 24747 |
| Unique reflections | 7928 | 7289 |
| reflections with $I > 2\sigma(I)$ | 4507 | 5848 |
| $R_{int}$ | - | 0.080 |
| Number of restraints | 18 | 238 |
| Parameters | 493 | 635 |
| $R1$ [$I>2\sigma(I)$] | 0.0641 | 0.0692 |
| $wR2$ [$I>2\sigma(I)$] | 0.1418 | 0.1814 |
| $R1$ [all data] | 0.1285 | 0.0843 |
| $wR2$ [all data] | 0.1721 | 0.1930 |
| GooF | 1.03 | 1.031 |
| Extinction coefficient | | |
| Largest diff. peak / hole max. / min. [$10^3 \cdot e \cdot nm^{-3}$] | 0.31 and -0.31 | 0.485 and -0.335 |

See Reference S3 for single crystal X-ray data of compound **2b** at 23 K. It is given here for proper comparison.

**Table S4.** Bond lengths [Å] and angles [°] for $(Me_2\text{-cAAC})_2Si_2$ (**2a**).

| | | | |
|---|---|---|---|
| Si1A—C1 | 1.887 (4) | C4B—C5B | 1.48 (6) |
| Si1A—Si1A$^i$ | 2.254 (2) | C5B—C9B | 1.42 (5) |
| Si1B—C1 | 1.978 (6) | C5B—C8B | 1.51 (5) |
| Si1B—Si1B$^i$ | 2.232 (9) | C10—C15 | 1.406 (5) |
| C1—N2 | 1.342 (5) | C10—C11 | 1.416 (5) |
| C1—C5A | 1.502 (15) | C11—C12 | 1.395 (5) |
| C1—C5B | 1.64 (5) | C11—C16 | 1.520 (5) |
| N2—C10 | 1.453 (4) | C12—C13 | 1.375 (6) |
| N2—C3 | 1.504 (4) | C13—C14 | 1.372 (6) |
| C3—C6B | 1.31 (4) | C14—C15 | 1.395 (5) |
| C3—C7 | 1.517 (6) | C15—C19 | 1.527 (6) |

| | | | |
|---|---|---|---|
| C3—C4A | 1.534 (8) | C16—C17 | 1.521 (7) |
| C3—C4B | 1.56 (3) | C16—C18B | 1.53 (2) |
| C3—C6A | 1.568 (10) | C16—C18A | 1.561 (8) |
| C4A—C5A | 1.546 (18) | C19—C21A | 1.527 (7) |
| C5A—C8A | 1.532 (15) | C19—C20 | 1.53.0 (6) |
| C5A—C9A | 1.567 (12) | C19—C21B | 1.64 (2) |
| C1—Si1A—Si1A$^{i}$ | 103.67 (14) | C5B—C4B—C3 | 109 (3) |
| C1—Si1B—Si1B$^{i}$ | 98.9 (3) | C9B—C5B—C4B | 123 (3) |
| N2—C1—C5A | 109.0 (7) | C9B—C5B—C8B | 108 (4) |
| N2—C1—C5B | 105.9 (19) | C4B—C5B—C8B | 104 (4) |
| N2—C1—Si1A | 122.6 (3) | C9B—C5B—C1 | 119 (4) |
| C5A—C1—Si1A | 126.3 (6) | C4B—C5B—C1 | 104 (3) |
| N2—C1—Si1B | 119.7 (3) | C8B—C5B—C1 | 95 (2) |
| C5B—C1—Si1B | 118 (2) | C15—C10—C11 | 120.8 (3) |
| C1—N2—C10 | 121.9 (3) | C15—C10—N2 | 120.3 (3) |
| C1—N2—C3 | 115.5 (3) | C11—C10—N2 | 118.9 (3) |
| C10—N2—C3 | 122.6 (3) | C12—C11—C10 | 118.1 (3) |
| C6B—C3—N2 | 112.8 (19) | C12—C11—C16 | 118.7 (4) |
| C6B—C3—C7 | 116.9 (18) | C10—C11—C16 | 123.1 (3) |
| N2—C3—C7 | 112.2 (3) | C13—C12—C11 | 121.3 (4) |
| N2—C3—C4A | 99.8 (4) | C14—C13—C12 | 120.0 (4) |
| C7—C3—C4A | 116.5 (4) | C13—C14—C15 | 121.7 (4) |
| C6B—C3—C4B | 112 (2) | C14—C15—C10 | 118.0 (4) |
| N2—C3—C4B | 102.9 (12) | C14—C15—C19 | 117.5 (3) |
| C7—C3—C4B | 98.2 (11) | C10—C15—C19 | 124.5 (3) |
| N2—C3—C6A | 111.5 (6) | C11—C16—C17 | 111.5 (4) |
| C7—C3—C6A | 106.9 (5) | C11—C16—C18B | 122.7 (9) |

| | | | |
|---|---|---|---|
| C4A—C3—C6A | 110.0 (5) | C17—C16—C18B | 88.1 (9) |
| C3—C4A—C5A | 107.5 (7) | C11—C16—C18A | 107.8 (4) |
| C1—C5A—C8A | 118.4 (8) | C17—C16—C18A | 114.6 (4) |
| C1—C5A—C4A | 102.4 (9) | C15—C19—C21A | 113.0 (4) |
| C8A—C5A—C4A | 111.2 (10) | C15—C19—C20 | 112.1 (3) |
| C1—C5A—C9A | 105.4 (9) | C21A—C19—C20 | 105.0 (4) |
| C8A—C5A—C9A | 108.6 (10) | C15—C19—C21B | 100.6 (8) |
| C4A—C5A—C9A | 110.5 (7) | C20—C19—C21B | 129.6 (8) |

Symmetry code: (i) –x+1, -y+2, -z+1.

**Table S5.** Bond lengths [Å] and angles [°] for (Cy-cAAC)$_2$Si$_2$ (**2b**) (at T = 100 K).

| | | | |
|---|---|---|---|
| Si1A—C1A | 1.878 (5) | C16A—C17A | 1.372 (5) |
| Si1A—Si2A | 2.255 (4) | C17A—C18A | 1.397 (5) |
| Si2A—C1C | 1.861 (4) | C18A—C19A | 1.517 (5) |
| Si1C—C1B | 1.836 (18) | C19A—C20A | 1.519 (6) |
| Si1C—Si2C | 2.232 (9) | C19A—C21A | 1.544 (5) |
| Si2C—C1C | 2.016 (6) | C22A—C24A | 1.535 (6) |
| C1A—N2A | 1.347 (6) | C22A—C23A | 1.541 (6) |
| C1A—C5A | 1.534 (6) | C22B—C23B | 1.541 (10) |
| N2A—C13A | 1.440 (5) | C22B—C24B | 1.549 (10) |
| N2A—C3A | 1.512 (7) | C1C—N2C | 1.349 (5) |
| C3A—C7A | 1.525 (6) | C1C—C5C | 1.527 (5) |
| C3A—C4A | 1.533 (7) | N2C—C13C | 1.445 (4) |
| C3A—C6A | 1.537 (6) | N2C—C3C | 1.506 (4) |
| C4A—C5A | 1.532 (6) | C3C—C7C | 1.503 (5) |
| C5A—C12A | 1.538 (7) | C3C—C6C | 1.513 (5) |
| C5A—C8A | 1.549 (10) | C3C—C4D | 1.516 (13) |
| C8A—C9A | 1.540 (6) | C3C—C4C | 1.568 (10) |
| C9A—C10A | 1.520 (7) | C4C—C5C | 1.550 (7) |
| C10A—C11A | 1.535 (7) | C4D—C5C | 1.610 (10) |
| C11A—C12A | 1.536 (6) | C5C—C12C | 1.521 (5) |
| C1B—N2B | 1.33 (2) | C5C—C8C | 1.524 (6) |

| | | | |
|---|---|---|---|
| C1B—C5B | 1.56 (3) | C8C—C9C | 1.549 (6) |
| N2B—C3B | 1.50 (3) | C9C—C10C | 1.479 (6) |
| N2B—C13A | 1.58 (2) | C10C—C11C | 1.493 (6) |
| C3B—C4B | 1.48 (3) | C11C—C12C | 1.525 (5) |
| C3B—C6B | 1.516 (11) | C13C—C18C | 1.400 (5) |
| C3B—C7B | 1.525 (10) | C13C—C14C | 1.413 (5) |
| C4B—C5B | 1.60 (3) | C14C—C15C | 1.406 (5) |
| C5B—C8B | 1.44 (4) | C14C—C22C | 1.518 (5) |
| C5B—C12B | 1.543 (11) | C15C—C16C | 1.368 (5) |
| C8B—C9B | 1.540 (10) | C16C—C17C | 1.375 (5) |
| C9B—C10B | 1.530 (11) | C17C—C18C | 1.405 (5) |
| C10B—C11B | 1.535 (10) | C18C—C19D | 1.523 (10) |
| C11B—C12B | 1.542 (10) | C18C—C19C | 1.527 (5) |
| C13A—C14A | 1.404 (5) | C19C—C20C | 1.531 (7) |
| C13A—C18A | 1.416 (5) | C19C—C21C | 1.550 (7) |
| C14A—C15A | 1.400 (5) | C19D—C20D | 1.52 (4) |
| C14A—C22A | 1.517 (5) | C19D—C21D | 1.60 (3) |
| C14A—C22B | 1.542 (10) | C22C—C23C | 1.533 (5) |
| C15A—C16A | 1.372 (5) | C22C—C24C | 1.536 (5) |
| | | | |
| C1A—Si1A—Si2A | 101.99 (14) | C13A—C18A—C19A | 123.8 (3) |
| C1C—Si2A—Si1A | 104.24 (12) | C18A—C19A—C20A | 113.9 (3) |
| C1B—Si1C—Si2C | 105.1 (6) | C18A—C19A—C21A | 109.1 (3) |
| C1C—Si2C—Si1C | 97.9 (3) | C20A—C19A—C21A | 109.8 (3) |
| N2A—C1A—C5A | 107.9 (3) | C14A—C22A—C24A | 110.5 (3) |
| N2A—C1A—Si1A | 119.3 (3) | C14A—C22A—C23A | 113.3 (4) |
| C5A—C1A—Si1A | 132.3 (3) | C24A—C22A—C23A | 108.2 (4) |
| C1A—N2A—C13A | 123.5 (3) | C23B—C22B—C14A | 108.1 (15) |
| C1A—N2A—C3A | 115.7 (4) | C23B—C22B—C24B | 110.9 (18) |
| C13A—N2A—C3A | 120.6 (4) | C14A—C22B—C24B | 113.6 (15) |
| N2A—C3A—C7A | 112.1 (5) | N2C—C1C—C5C | 108.8 (2) |
| N2A—C3A—C4A | 100.7 (4) | N2C—C1C—Si2A | 124.5 (2) |
| C7A—C3A—C4A | 112.1 (4) | C5C—C1C—Si2A | 124.8 (3) |
| N2A—C3A—C6A | 111.0 (4) | N2C—C1C—Si2C | 111.1 (3) |
| C7A—C3A—C6A | 109.1 (5) | C5C—C1C—Si2C | 133.3 (3) |
| C4A—C3A—C6A | 111.7 (4) | C1C—N2C—C13C | 122.3 (2) |

| | | | |
|---|---|---|---|
| C5A—C4A—C3A | 107.7 (4) | C1C—N2C—C3C | 115.7 (2) |
| C4A—C5A—C1A | 104.0 (3) | C13C—N2C—C3C | 122.0 (3) |
| C4A—C5A—C12A | 111.6 (4) | C7C—C3C—N2C | 110.9 (3) |
| C1A—C5A—C12A | 112.8 (4) | C7C—C3C—C6C | 108.7 (3) |
| C4A—C5A—C8A | 112.0 (4) | N2C—C3C—C6C | 111.5 (3) |
| C1A—C5A—C8A | 108.4 (4) | C7C—C3C—C4D | 97.7 (6) |
| C12A—C5A—C8A | 108.1 (4) | N2C—C3C—C4D | 102.1 (4) |
| C9A—C8A—C5A | 113.3 (6) | C6C—C3C—C4D | 124.9 (6) |
| C10A—C9A—C8A | 111.2 (6) | C7C—C3C—C4C | 121.9 (6) |
| C9A—C10A—C11A | 111.6 (5) | N2C—C3C—C4C | 100.8 (3) |
| C10A—C11A—C12A | 109.8 (4) | C6C—C3C—C4C | 102.6 (5) |
| C11A—C12A—C5A | 112.1 (4) | C5C—C4C—C3C | 106.2 (5) |
| N2B—C1B—C5B | 108.8 (15) | C3C—C4D—C5C | 105.8 (7) |
| N2B—C1B—Si1C | 127.1 (14) | C12C—C5C—C8C | 108.7 (3) |
| C5B—C1B—Si1C | 120.9 (12) | C12C—C5C—C1C | 110.7 (2) |
| C1B—N2B—C3B | 117.5 (17) | C8C—C5C—C1C | 110.7 (3) |
| C1B—N2B—C13A | 122.8 (15) | C12C—C5C—C4C | 120.1 (5) |
| C3B—N2B—C13A | 118.3 (14) | C8C—C5C—C4C | 101.9 (5) |
| C4B—C3B—N2B | 101.0 (16) | C1C—C5C—C4C | 104.3 (4) |
| C4B—C3B—C6B | 115.7 (19) | C12C—C5C—C4D | 98.3 (6) |
| N2B—C3B—C6B | 109.1 (18) | C8C—C5C—C4D | 125.3 (6) |
| C4B—C3B—C7B | 115 (2) | C1C—C5C—C4D | 102.2 (5) |
| N2B—C3B—C7B | 109 (2) | C5C—C8C—C9C | 111.4 (3) |
| C6B—C3B—C7B | 107 (2) | C10C—C9C—C8C | 111.7 (3) |
| C3B—C4B—C5B | 110.3 (17) | C9C—C10C—C11C | 112.2 (4) |
| C8B—C5B—C12B | 116 (2) | C10C—C11C—C12C | 111.3 (3) |
| C8B—C5B—C1B | 119.7 (18) | C5C—C12C—C11C | 112.3 (3) |
| C12B—C5B—C1B | 103.9 (15) | C18C—C13C—C14C | 120.9 (3) |
| C8B—C5B—C4B | 109 (2) | C18C—C13C—N2C | 120.0 (3) |
| C12B—C5B—C4B | 106.0 (18) | C14C—C13C—N2C | 119.1 (3) |
| C1B—C5B—C4B | 99.9 (15) | C15C—C14C—C13C | 117.8 (3) |
| C5B—C8B—C9B | 112 (3) | C15C—C14C—C22C | 117.7 (3) |
| C10B—C9B—C8B | 111 (3) | C13C—C14C—C22C | 124.4 (3) |
| C9B—C10B—C11B | 113 (3) | C16C—C15C—C14C | 121.5 (3) |
| C10B—C11B—C12B | 108 (3) | C15C—C16C—C17C | 119.8 (3) |
| C11B—C12B—C5B | 115 (2) | C16C—C17C—C18C | 121.6 (3) |

| | | | |
|---|---|---|---|
| C14A—C13A—C18A | 121.6 (3) | C13C—C18C—C17C | 117.9 (3) |
| C14A—C13A—N2A | 117.1 (3) | C13C—C18C—C19D | 133.7 (12) |
| C18A—C13A—N2A | 121.3 (3) | C17C—C18C—C19D | 107.3 (12) |
| C14A—C13A—N2B | 133.8 (6) | C13C—C18C—C19C | 122.3 (4) |
| C18A—C13A—N2B | 104.5 (7) | C17C—C18C—C19C | 119.6 (4) |
| C15A—C14A—C13A | 117.4 (3) | C18C—C19C—C20C | 111.3 (5) |
| C15A—C14A—C22A | 116.8 (3) | C18C—C19C—C21C | 109.4 (5) |
| C13A—C14A—C22A | 125.7 (3) | C20C—C19C—C21C | 108.1 (4) |
| C15A—C14A—C22B | 130.5 (10) | C20D—C19D—C18C | 113 (2) |
| C13A—C14A—C22B | 110.1 (10) | C20D—C19D—C21D | 109.1 (19) |
| C16A—C15A—C14A | 121.5 (3) | C18C—C19D—C21D | 119.2 (19) |
| C15A—C16A—C17A | 120.5 (3) | C14C—C22C—C23C | 112.9 (3) |
| C16A—C17A—C18A | 121.3 (3) | C14C—C22C—C24C | 110.4 (3) |
| C17A—C18A—C13A | 117.4 (3) | C23C—C22C—C24C | 108.1 (3) |
| C17A—C18A—C19A | 118.5 (3) | | |

**Table S6.** Bond lengths [Å] and angles [°] for $(Me_2\text{-cAAC})_2Si_2Se_4\cdot 2THF$ (**3a·2THF**)

| | | | |
|---|---|---|---|
| Se2—Si1 | 2.1510 (10) | C17—H17A | 0.9800 |
| Se1—Si1 | 2.2874 (10) | C17—H17B | 0.9800 |
| Se1—Si1$^{i}$ | 2.3045 (10) | C17—H17C | 0.9800 |
| Si1—C1 | 1.931 (4) | C18—C20 | 1.525 (5) |
| Si1—Se1$^{i}$ | 2.3046 (10) | C18—C19 | 1.538 (5) |
| N1—C1 | 1.311 (5) | C18—H18 | 1.0000 |
| N1—C9 | 1.464 (5) | C19—H19A | 0.9800 |
| N1—C4 | 1.540 (4) | C19—H19B | 0.9800 |
| C1—C2 | 1.528 (5) | C19—H19C | 0.9800 |
| C2—C3A | 1.516 (8) | C20—H20A | 0.9800 |
| C2—C5 | 1.521 (6) | C20—H20B | 0.9800 |
| C2—C6 | 1.527 (6) | C20—H20C | 0.9800 |
| C2—C3B | 1.599 (12) | C3A—H3A | 0.9900 |
| C4—C3B | 1.410 (13) | C3A—H3B | 0.9900 |
| C4—C8A | 1.444 (8) | C7A—H7AA | 0.9800 |
| C4—C7B | 1.469 (12) | C7A—H7AB | 0.9800 |
| C4—C3A | 1.555 (9) | C7A—H7AC | 0.9800 |
| C4—C7A | 1.615 (8) | C8A—H8A1 | 0.9800 |

| | | | |
|---|---|---|---|
| C4—C8B | 1.650 (12) | C8A—H8A2 | 0.9800 |
| C5—H5A | 0.9800 | C8A—H8A3 | 0.9800 |
| C5—H5B | 0.9800 | C3B—H3B1 | 0.9900 |
| C5—H5C | 0.9800 | C3B—H3B2 | 0.9900 |
| C6—H6A | 0.9800 | C7B—H7BA | 0.9800 |
| C6—H6B | 0.9800 | C7B—H7BB | 0.9800 |
| C6—H6C | 0.9800 | C7B—H7BC | 0.9800 |
| C9—C10 | 1.407 (5) | C8B—H8B1 | 0.9800 |
| C9—C14 | 1.410 (5) | C8B—H8B2 | 0.9800 |
| C10—C11 | 1.401 (6) | C8B—H8B3 | 0.9800 |
| C10—C15 | 1.525 (5) | O5T_1—C1T_1 | 1.395 (8) |
| C11—C12 | 1.377 (6) | O5T_1—C4T_1 | 1.411 (7) |
| C11—H11 | 0.9500 | C1T_1—C2T_1 | 1.453 (8) |
| C12—C13 | 1.384 (6) | C1T_1—H1TA_1 | 0.9900 |
| C12—H12 | 0.9500 | C1T_1—H1TB_1 | 0.9900 |
| C13—C14 | 1.386 (5) | C2T_1—C3T_1 | 1.478 (9) |
| C13—H13 | 0.9500 | C2T_1—H2TA_1 | 0.9900 |
| C14—C18 | 1.522 (5) | C2T_1—H2TB_1 | 0.9900 |
| C15—C16 | 1.527 (5) | C3T_1—C4T_1 | 1.503 (8) |
| C15—C17 | 1.528 (6) | C3T_1—H3TA_1 | 0.9900 |
| C15—H15 | 1.0000 | C3T_1—H3TB_1 | 0.9900 |
| C16—H16A | 0.9800 | C4T_1—H4TA_1 | 0.9900 |
| C16—H16B | 0.9800 | C4T_1—H4TB_1 | 0.9900 |
| C16—H16C | 0.9800 | | |
| Si1—Se1—Si1$^{i}$ | 83.45 (4) | H17B—C17—H17C | 109.5 |
| C1—Si1—Se2 | 97.86 (11) | C14—C18—C20 | 110.0 (3) |
| C1—Si1—Se1 | 113.81 (11) | C14—C18—C19 | 112.3 (3) |
| Se2—Si1—Se1 | 120.35 (4) | C20—C18—C19 | 110.1 (3) |
| C1—Si1—Se1$^{i}$ | 109.85 (11) | C14—C18—H18 | 108.1 |
| Se2—Si1—Se1$^{i}$ | 119.05 (4) | C20—C18—H18 | 108.1 |
| Se1—Si1—Se1$^{i}$ | 96.55 (4) | C19—C18—H18 | 108.1 |
| C1—N1—C9 | 125.6 (3) | C18—C19—H19A | 109.5 |
| C1—N1—C4 | 113.2 (3) | C18—C19—H19B | 109.5 |
| C9—N1—C4 | 121.0 (3) | H19A—C19—H19B | 109.5 |
| N1—C1—C2 | 110.0 (3) | C18—C19—H19C | 109.5 |
| N1—C1—Si1 | 126.9 (3) | H19A—C19—H19C | 109.5 |

| | | | |
|---|---|---|---|
| C2—C1—Si1 | 122.3 (3) | H19B—C19—H19C | 109.5 |
| C3A—C2—C5 | 120.1 (5) | C18—C20—H20A | 109.5 |
| C3A—C2—C6 | 103.4 (5) | C18—C20—H20B | 109.5 |
| C5—C2—C6 | 107.1 (4) | H20A—C20—H20B | 109.5 |
| C3A—C2—C1 | 102.9 (4) | C18—C20—H20C | 109.5 |
| C5—C2—C1 | 107.8 (3) | H20A—C20—H20C | 109.5 |
| C6—C2—C1 | 116.0 (3) | H20B—C20—H20C | 109.5 |
| C5—C2—C3B | 98.1 (6) | C2—C3A—C4 | 106.4 (5) |
| C6—C2—C3B | 125.5 (6) | C2—C3A—H3A | 110.5 |
| C1—C2—C3B | 100.1 (5) | C4—C3A—H3A | 110.5 |
| C3B—C4—C7B | 124.1 (8) | C2—C3A—H3B | 110.5 |
| C3B—C4—N1 | 101.3 (5) | C4—C3A—H3B | 110.5 |
| C8A—C4—N1 | 112.7 (4) | H3A—C3A—H3B | 108.6 |
| C7B—C4—N1 | 111.7 (5) | C4—C7A—H7AA | 109.5 |
| C8A—C4—C3A | 115.7 (5) | C4—C7A—H7AB | 109.5 |
| N1—C4—C3A | 101.1 (4) | H7AA—C7A—H7AB | 109.5 |
| C8A—C4—C7A | 107.4 (5) | C4—C7A—H7AC | 109.5 |
| N1—C4—C7A | 110.3 (4) | H7AA—C7A—H7AC | 109.5 |
| C3A—C4—C7A | 109.5 (5) | H7AB—C7A—H7AC | 109.5 |
| C3B—C4—C8B | 107.8 (8) | C4—C8A—H8A1 | 109.5 |
| C7B—C4—C8B | 106.6 (7) | C4—C8A—H8A2 | 109.5 |
| N1—C4—C8B | 103.6 (5) | H8A1—C8A—H8A2 | 109.5 |
| C2—C5—H5A | 109.5 | C4—C8A—H8A3 | 109.5 |
| C2—C5—H5B | 109.5 | H8A1—C8A—H8A3 | 109.5 |
| H5A—C5—H5B | 109.5 | H8A2—C8A—H8A3 | 109.5 |
| C2—C5—H5C | 109.5 | C4—C3B—C2 | 109.5 (8) |
| H5A—C5—H5C | 109.5 | C4—C3B—H3B1 | 109.8 |
| H5B—C5—H5C | 109.5 | C2—C3B—H3B1 | 109.8 |
| C2—C6—H6A | 109.5 | C4—C3B—H3B2 | 109.8 |
| C2—C6—H6B | 109.5 | C2—C3B—H3B2 | 109.8 |
| H6A—C6—H6B | 109.5 | H3B1—C3B—H3B2 | 108.2 |
| C2—C6—H6C | 109.5 | C4—C7B—H7BA | 109.5 |
| H6A—C6—H6C | 109.5 | C4—C7B—H7BB | 109.5 |
| H6B—C6—H6C | 109.5 | H7BA—C7B—H7BB | 109.5 |
| C10—C9—C14 | 121.6 (3) | C4—C7B—H7BC | 109.5 |
| C10—C9—N1 | 121.5 (3) | H7BA—C7B—H7BC | 109.5 |

| | | | |
|---|---|---|---|
| C14—C9—N1 | 116.9 (3) | H7BB—C7B—H7BC | 109.5 |
| C11—C10—C9 | 117.3 (4) | C4—C8B—H8B1 | 109.5 |
| C11—C10—C15 | 117.0 (4) | C4—C8B—H8B2 | 109.5 |
| C9—C10—C15 | 125.7 (4) | H8B1—C8B—H8B2 | 109.5 |
| C12—C11—C10 | 122.0 (4) | C4—C8B—H8B3 | 109.5 |
| C12—C11—H11 | 119.0 | H8B1—C8B—H8B3 | 109.5 |
| C10—C11—H11 | 119.0 | H8B2—C8B—H8B3 | 109.5 |
| C11—C12—C13 | 119.3 (4) | C1T_1—O5T_1—C4T_1 | 105.8 (5) |
| C11—C12—H12 | 120.4 | O5T_1—C1T_1—C2T_1 | 111.7 (6) |
| C13—C12—H12 | 120.4 | O5T_1—C1T_1—H1TA_1 | 109.3 |
| C12—C13—C14 | 121.9 (4) | C2T_1—C1T_1—H1TA_1 | 109.3 |
| C12—C13—H13 | 119.1 | O5T_1—C1T_1—H1TB_1 | 109.3 |
| C14—C13—H13 | 119.1 | C2T_1—C1T_1—H1TB_1 | 109.3 |
| C13—C14—C9 | 117.9 (4) | H1TA_1—C1T_1—H1TB_1 | 108.0 |
| C13—C14—C18 | 117.3 (3) | C1T_1—C2T_1—C3T_1 | 104.5 (6) |
| C9—C14—C18 | 124.7 (3) | C1T_1—C2T_1—H2TA_1 | 110.8 |
| C10—C15—C16 | 110.8 (3) | C3T_1—C2T_1—H2TA_1 | 110.8 |
| C10—C15—C17 | 110.9 (3) | C1T_1—C2T_1—H2TB_1 | 110.8 |
| C16—C15—C17 | 108.9 (4) | C3T_1—C2T_1—H2TB_1 | 110.8 |
| C10—C15—H15 | 108.7 | H2TA_1—C2T_1—H2TB_1 | 108.9 |
| C16—C15—H15 | 108.7 | C2T_1—C3T_1—C4T_1 | 104.4 (6) |
| C17—C15—H15 | 108.7 | C2T_1—C3T_1—H3TA_1 | 110.9 |
| C15—C16—H16A | 109.5 | C4T_1—C3T_1—H3TA_1 | 110.9 |
| C15—C16—H16B | 109.5 | C2T_1—C3T_1—H3TB_1 | 110.9 |
| H16A—C16—H16B | 109.5 | C4T_1—C3T_1—H3TB_1 | 110.9 |
| C15—C16—H16C | 109.5 | H3TA_1—C3T_1—H3TB_1 | 108.9 |
| H16A—C16—H16C | 109.5 | O5T_1—C4T_1—C3T_1 | 107.0 (5) |
| H16B—C16—H16C | 109.5 | O5T_1—C4T_1—H4TA_1 | 110.3 |
| C15—C17—H17A | 109.5 | C3T_1—C4T_1—H4TA_1 | 110.3 |
| C15—C17—H17B | 109.5 | O5T_1—C4T_1—H4TB_1 | 110.3 |
| H17A—C17—H17B | 109.5 | C3T_1—C4T_1—H4TB_1 | 110.3 |
| C15—C17—H17C | 109.5 | H4TA_1—C4T_1—H4TB_1 | 108.6 |
| H17A—C17—H17C | 109.5 | | |

Symmetry Code: (i) –x+1, -y+1, -z.

**Table S7.** Crystal data and structure refinement for $Me_2$-cAAC=O.

| Compound | $Me_2$-cAAC=O |
|---|---|
| Empirical formula | $C_{20}H_{31}NO$ |
| CCDC no. | 927696 |
| Molecular weight | 301.46 |
| Crystal size [mm] | 0.16 × 0.14 × 0.10 |
| Wavelength [pm] | 1.54188 |
| Crystal system | Monoclinic |
| Space group | $P2_1/n$ |
| $a$ [Å] | 9.269(7) |
| $b$ [Å] | 17.342(10) |
| $c$ [Å] | 11.775(11) |
| $\alpha$ [°] | 90.0 |
| $\beta$ [°] | 96.71(4) |
| $\gamma$ [°] | 90.0 |
| $V$ [Å $^3$] | 1880(2) |
| $Z$ | 4 |
| Temperature [K] | 100(2) |
| $\rho$ [Mgm$^{-3}$] | 1.065 |
| $\mu$ [mm$^{-1}$] | 1.54188 |
| $F$ (000) | 664 |
| $\theta$-area [°] | 4.6 to 69.5 |
| Total number reflect. | 30611 |
| Unique reflections | 3254 |
| $R_{int}$ | 0.0353 |
| Number of restraints | 0 |
| Parameters | 230 |
| $R1$ [$I>2\sigma(I)$] | 0.024 |
| $wR2$ [$I>2\sigma(I)$] | 0.021 |
| $R1$ [all data] | 0.027 |
| $wR2$ [all data] | 0.021 |
| GooF | 2.19 |
| Extinction coefficient | |
| Largest diff. peak / hole max. / min. [$10^3\cdot$e$\cdot$nm$^{-3}$] | 0.0198 and -0.127 |

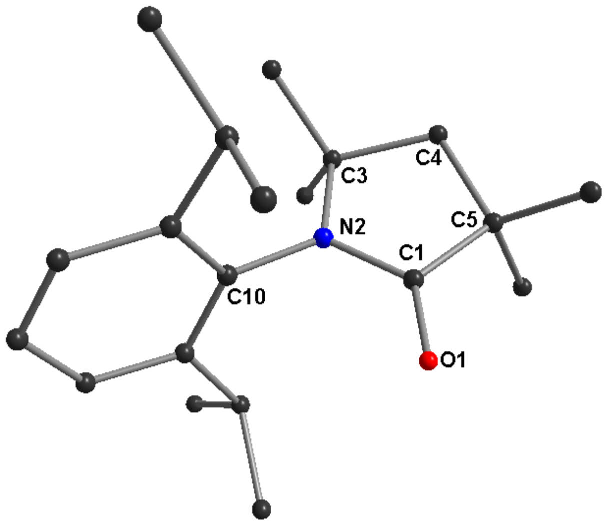


**Figure S9**. Crystal structure of $Me_2$-cAAC=O.

**Table S8.** Bond lengths [Å] and angles [°] for $Me_2$-cAAC=O.

| | | | |
|---|---|---|---|
| O(1)—C(1) | 1.2386 (10) | C(12)—C(13) | 1.4059 (12) |
| N(2)—C(1) | 1.3736 (10) | C(12)—H(12) | 1.0823 |
| N(2)—C(10) | 1.4482 (10) | C(13)—C(14) | 1.4058 (12) |
| C(4)—H(4A) | 1.0937 | C(13)—H(13) | 1.0822 |
| C(4)—H(4B) | 1.0937 | C(14)—C(15) | 1.4118 (11) |
| C(6)—H(6A) | 1.0914 | C(14)—H(14) | 1.0822 |
| C(6)—H(6B) | 1.0914 | C(15)—C(19) | 1.5367 (11) |
| C(6)—H(6C) | 1.0914 | C(16)—H(16) | 1.0939 |
| C(7)—H(7A) | 1.0914 | C(17)—H(17A) | 1.0914 |
| C(7)—H(7B) | 1.0914 | C(17)—H(17B) | 1.0914 |
| C(7)—H(7C) | 1.0914 | C(17)—H(17C) | 1.0914 |
| C(8)—H(8A) | 1.0914 | C(18)—H(18A) | 1.0914 |
| C(8)—H(8B) | 1.0914 | C(18)—H(18B) | 1.0914 |
| C(8)—H(8C) | 1.0914 | C(18)—H(18C) | 1.0914 |
| C(9)—H(9A) | 1.0914 | C(19)—H(19) | 1.0939 |
| C(9)—H(9B) | 1.0914 | C(20)—H(20A) | 1.0914 |

| | | | |
|---|---|---|---|
| C(9)—H(9C) | 1.0914 | C(20)—H(20B) | 1.0914 |
| C(10)—C(11) | 1.4292 (11) | C(20)—H(20C) | 1.0914 |
| C(10)—C(15) | 1.4270 (11) | C(21)—H(21A) | 1.0914 |
| C(11)—C(12) | 1.4123 (11) | C(21)—H(21B) | 1.0914 |
| C(11)—C(16) | 1.5363 (12) | C(21)—H(21C) | 1.0914 |
| C(1)—N(2)—C(10) | 122.73 (6) | C(12)—C(11)—C(16) | 119.15 (7) |
| O(1)—C(1)—N(2) | 125.44 (7) | C(11)—C(12)—C(13) | 121.08 (8) |
| N(2)—C(10)—C(11) | 118.98 (7) | C(12)—C(13)—C(14) | 119.70 (8) |
| N(2)—C(10)—C(15) | 120.07 (7) | C(13)—C(14)—C(15) | 121.33 (8) |
| C(11)—C(10)—C(15) | 120.95 (7) | C(10)—C(15)—C(14) | 118.20 (7) |
| C(10)—C(11)—C(12) | 118.37 (8) | C(10)—C(15)—C(19) | 122.81 (7) |
| C(10)—C(11)—C(16) | 122.30 (7) | C(14)—C(15)—C(19) | 118.79 (7) |

### (S4) Solid state NMR

All the solid-state experiments on compound **2** were done at a field strength of 9.4 T (400 MHz) using an Avance spectrometer (Bruker Biospin, Germany) at room temperature. $^{29}$Si CP MAS NMR spectra were acquired using a 2.5 mm triple channel probe whereas $^{13}$C CPMAS NMR spectra were acquired with a 4 mm triple channel probe. The carbon spectra were calibrated by referencing externally to the $^{13}$CH resonance (31.46 ppm) of solid adamantane. Similarly, $^{29}$Si spectra were externally referenced to the single silicon resonance (0 ppm) of sodium salt of trimethyl-slilyl-propanoic acid. For silicon, the spectra were acquired at two spinning speeds of 7 and 18 kHz to determine the isotropic chemical shifts as shown in Figure SB for compound **2**. In a similar fashion, the carbon spectra were recorded at 8 kHz and 11 kHz MAS. Since, the compounds are air-sensitive; the samples were filled into the rotors in a glove box.

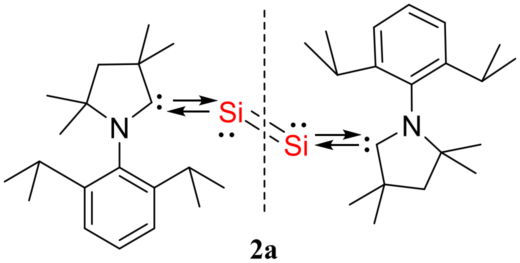

**2a**

**center of inversion (*i*)** even at 298 K;
Two Si-atoms are equivalent

**Solid state**: $^{29}$Si resonance 254.6 ppm
**Solution**: $^{29}$Si resonance 252.3 ppm

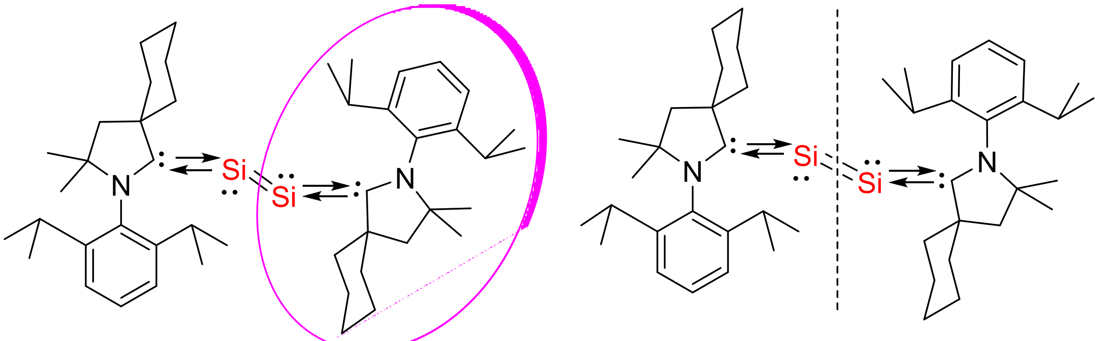

**No center of inversion (*i*)** even at 100 K;
Two Si-atoms are not equivalent

**center of inversion (*i*)** at 23 K;
Both Si-atoms are equivalent

**2b**

**Solid state**: $^{29}$Si resonances 190.1 and 318.3 ppm
**Solution**: $^{29}$Si resonance 249.1 ppm

**Scheme S6**: In $(Me_2\text{-cAAC})_2Si_2$ (**2a**) (top) center of inversion is always present while in $(Cy\text{-cAAC})_2Si_2$ (**2b**) (bottom) the presence of center of inversion is dependent on temperature. It is concluded from temperature dependent X-ray single crystal diffractions and solid state NMR. Structural comparison between **2a** and **2b** clearly shows that the carbene moieties (at 100 K) are evenly disordered in **2a** but not in **2b** (Figures S5-S8). This is also clearly observed in solid state $^{13}$C and $^{29}$Si NMR (see below).

The $^{29}$Si NMR spectra of $(Me_2\text{-cAAC})_2Si_2$ (**2a**) and $(Cy\text{-cAAC})_2Si_2$ (**2b**) which are recorded in $C_6D_6$ show single resonances at $\delta$ = 252.6 ppm for **2a** and 249.1 ppm for **2b** , they are more downfield shifted than that of $(NHC)_{2Si}$ ($\delta$ = 224.5 ppm).[S10] The $^{29}$Si resonance at 249.1 ppm (**4**) in solution falls in between 190.2 and 318.3 ppm. The $^{13}$C NMR spectra of **2a** and **2b** exhibit a resonance at $\delta$ = 236.7 ppm for **2a** and 234.7 ppm for **2b** which are more upfield shifted than that of cAAC: ($\delta$ = 304.2 ppm for $Me_2$-cAAC: and 309.4 ppm for Cy-cAAC:)[S1] but more downfield than that of $(Me_2\text{-cAAC})_2Si$ ($\delta$ = 210.0 ppm).[S11] The NMR spectra of both the compounds in solution

suggest that two silicon atoms are equivalent in solution while in solid state they are not. Robinson et al. have proposed [S12a] that NHC: stabilized $P_2$ allotrope can have two canonical forms; NHC:→P-P←:NHC and NHC=P-P=NHC. The former conformer [S12a] was established as the predominating product based on the chemical shift values of $^{31}$P NMR while the latter one [S12b] was shown to be the only conformer cAAC=P-P=CAAc for the cAAC: analogue.

**Solid-state CPMAS NMR experiments:** All the solid-state experiments were done at either a field strength of 9.4 T ($^{1}$H, 400 MHz) or 14.1 T ($^{1}$H, 600 MHz) using Avance III HD spectrometer (Bruker Biospin, Germany). $^{29}$Si- and $^{13}$C CPMAS NMR spectra were acquired using either a 2.5 mm or a 4 mm triple channel probe. The carbon spectra were calibrated by referencing externally to the $^{13}$CH resonance (31.46 ppm) of solid adamantane. Similarly, $^{29}$Si spectra were externally referenced to the single silicon resonance (0 ppm) of sodium salt of trimethyl-slilyl-propanoic acid.  For both nuclei, the spectra were acquired at two spinning speeds to determine the isotropic chemical shifts. Since, the compounds are air-sensitive the samples were filled into the rotors in a glove box.

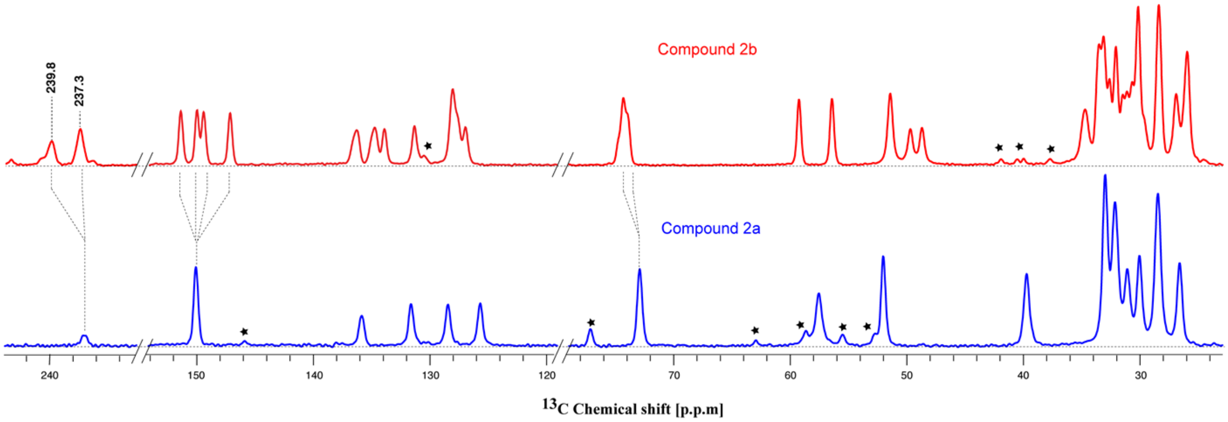


**Figure S10.** A comparison of solid-state $^{13}$C CP MAS spectra of compound **2a** and **2b** at a spinning speed of 11 kHz. The spinning sideband regions are denoted by ``*´´ symbol.  The carbene resonances are indicated in the figure. (T = 298 K)

**Compound 2a:** $^{13}$C (solid state NMR 298 K, $\delta$ ppm):  237.1 (C), 150.1, 135.8, 131.6, 128.4, 125.6, 72.9, 57,5, 52.0, 39.7, 32.1, 31.1, 30.1, 28.5, 26.6.

**Compound 2b:** $^{13}$C (solid state NMR 298 K, $\delta$ ppm):  239.8 (C), 237.3, 151.3, 149.9, 149.4, 147.1, 136.3, 134.7, 133.9, 131.3, 128.0, 127.6, 127.0, 74.3, 73.9, 59.2, 56.4, 51.4, 49.7, 48.6, 34.7, 33.5, 33.1, 32.6, 32.0, 31.5, 31.1, 30.7, 30.1, 29.6, 23.9, 26.9, 25.9.

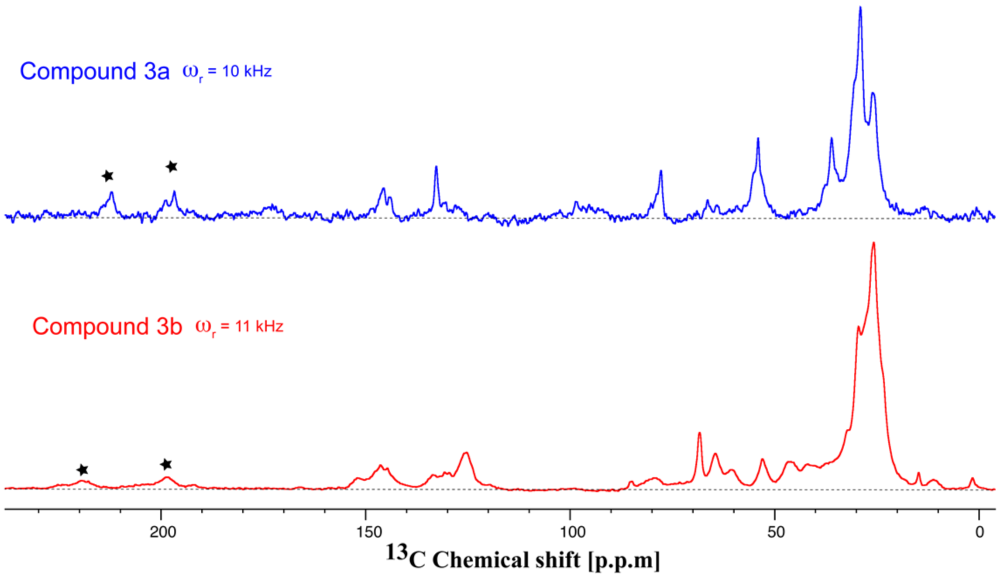


**Figure S11.** A comparison of solid-state $^{13}C$ CP MAS spectra of compound **3a** and **3b.** The spinning sideband regions are denoted by "★" symbol. The peaks are broader in comparison with the compound **2a** and **2b**. (T = 298 K)

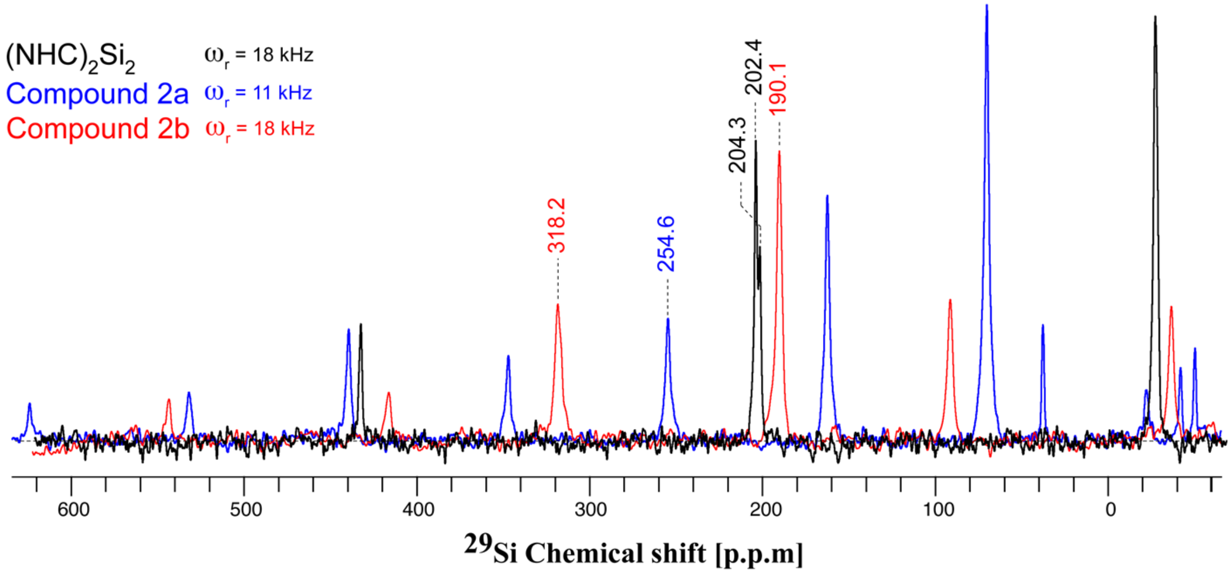


**Figure S12.** Comparison of Solid-state $^{29}Si$ CP MAS spectra of $(NHC)_2Si_2$, compound **2a**, and compound **2b.** Isotropic chemical shift values are indicated in the figure. All other peaks are spinning sidebands.

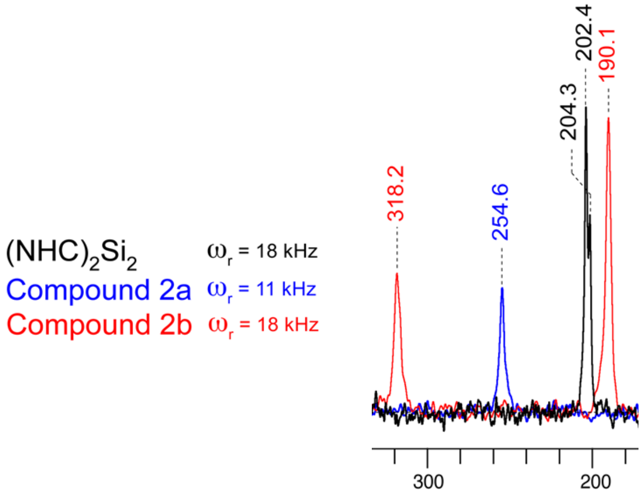


**Figure S13**. Solid state $^{29}$Si CPMAS NMR spectra of $(Me_2\text{-cAAC})_2Si_2$ (**2a**) (blue), $(Cy\text{-cAAC})_2Si_2$ (**2b**) (red) and $(NHC)_2Si_2$ (black).

### (S5) Theoretical calculations

### Computational Details:

All calculations were performed in Gaussian09 quantum package.[S13] Intermediates are optimized using the global-hybrid meta-GGA to DFT functional, R-M06-2X[S14] with SVP[S15] basis sets for all atoms. Geometries were fully optimized without symmetry constraints. Harmonic force constants were computed at the optimized geometries to confirm if they were located at minima or saddle point on the potential energy surface. For further validation of the energy change, we have also performed single point calculation of optimized geometry incorporating higher basis set TZVP[S16] for all atoms. Solvation energies in THF ($\varepsilon$ = 7.426) were evaluated by a self-consistent reaction field (SCRF) approach using the SMD continuum solvation model.[S17] NBO analysis were performed at R-M06-2X/TZVP//R-M06-2X/SVP level of theory. All the energy values reported in the manuscript are at R-M06-2X/TZVP/SMD//R-M06-2X/SVP level. The wavefunction file generated from the quantum code, were used to perform QTAIM[S18] analysis in the AIMALL program suite. We have applied Bader's AIM (Atoms-in-molecule)[S19] concept to characterize the electron distribution in **3a**. Any bonded pair of atoms has a bond path, *i.e;* a connecting line with maximum electron density. The bond critical point (BCP) is a point on this line where the gradient $\nabla\rho$(r) of the density is equal to zero. The magnitude of the electron density, $\rho$(r) and its Laplacian, $\nabla^2\rho$(r) at the BCP provide information about the strength and type of bond.

The Laplacian indicates whether the density is locally concentrated ($\nabla^2\rho<0$) or depleted ($\nabla^2\rho>0$). Optimized geometries and orbital diagrams are rendered in the Chemcraft visualization software.[S20]

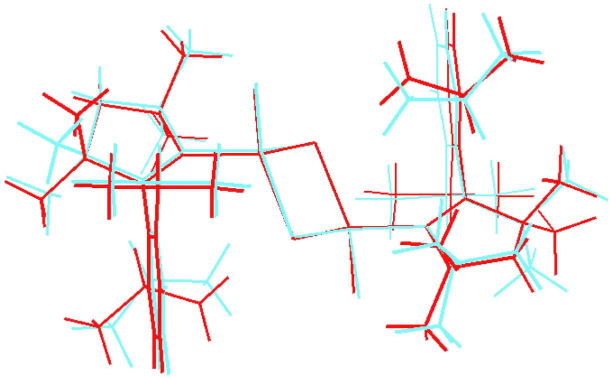

**Figure S14**. Superposition of crystal structure of **3a** with the optimized geometry at R-M06-2X/SVP level of theory.

**Table S9.** Topological parameters of important (3,-1) bond critical points (BCPs) of **3a**.

| Bond | $\rho$ (e/Å$^3$) | $\nabla^2\rho$ (e/Å$^5$) | DI |
|---|---|---|---|
| N1-C1 | 0.343 | -0.648 | 1.34 |
| C1-Si1 | 0.095 | +0.051 | 0.43 |
| Si1-Se1 | 0.083 | +0.009 | 0.58 |
| Si1-Se1´ | 0.083 | +0.005 | 0.60 |
| Si1-Se2 | 0.104 | +0.051 | 0.86 |

**Table S10.** Details Electronic Spectrum for the DFT optimized species **3a** at R-B3LYP/TZVP//R-M06-2X/SVP level. The cut-off on oscillator strength: 0.02. Total no. of states in the calculation: 20.

| λ (nm)[1] | $f^2$ | ΔE (cm$^{-1}$)[3] | Orbitals (percentage contribution)[4] |
|---|---|---|---|
| 441 | 0.030 | 22660.3. | H-1→L(73%); H-3→L(17%) |
| 430 | 0.029 | 23249.9 | H-1→L+1(86%); H-3→L+1(5%); |
| 418 | 0.028 | 23892.7 | H-2→L(73%); H-3→LUMO(12%) |
| 409 | 0.062 | 24421.8 | H-2→L+1(90%); H-2→L(6%) |

[1] Wavelength of the transition. [2] The oscillator strength of the transition. [3] Excitation energies for each transition. [4] Molecular orbitals involved in the transitions; H = HOMO, L = LUMO. The respective contributions are in parentheses.

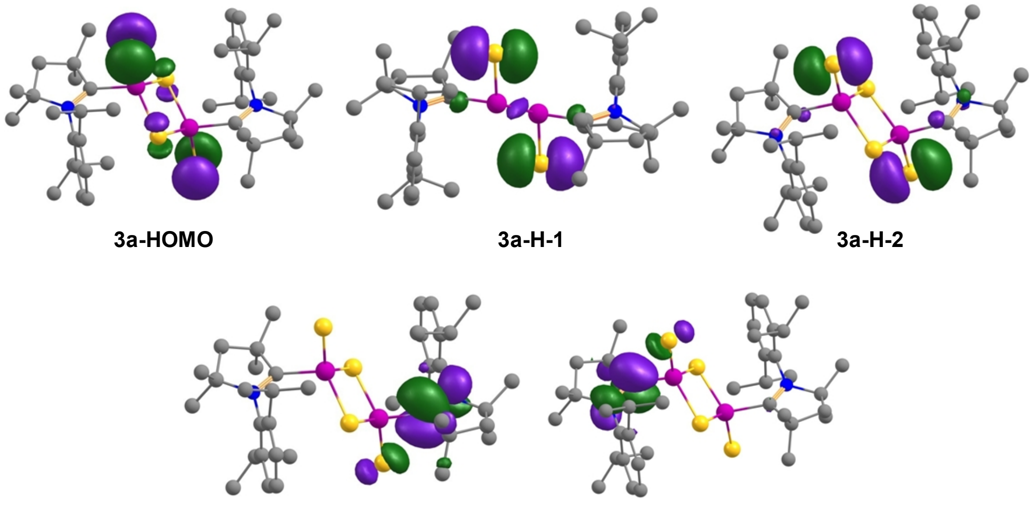


**Figure S15**. KS-MOs of some intermediates (isosurface = 0.05 au) and compound **3a**. Hydrogen atoms are omitted for clarity. H and L designate HOMO and LUMO.

**Table S11.** Calculated chemical shifts values (in ppm) of selected atoms for **3a** at R-B3LYP/TZVP//R-M06-2X/SVP level of theory.

| C1 | C1´ | Si1 | Si1´ | Se1 | Se1´ | Se2 | Se2´ |
|---|---|---|---|---|---|---|---|
| 209.4 | 207.5 | 12.4 | 18.2 | -536.8 | -526.6 | -637.9 | -600.6 |

**Table S12.** Cartesian coordinates (in Å) of the optimized structures of reactant, intermediates and product at R-M06-2X/SVP level of theory. Energy terms are in kcal/mol.

**2a**

Energy: -1410525.3847169

| | | | |
|---|---|---|---|
| Si | 0.11003 | 0.94383 | 0.59695 |
| N | -0.96236 | 3.33769 | -0.48777 |
| C | -2.69571 | 1.79606 | -0.32318 |
| C | -2.17599 | 4.17078 | -0.76854 |
| C | 0.98271 | 4.49002 | 0.50947 |
| C | 1.05019 | 3.76119 | -1.83949 |

| | | | |
|---|---|---|---|
| C | 0.36762 | 3.87700 | -0.60691 |
| C | 0.52008 | 2.97091 | -3.02716 |
| C | 2.31337 | 4.34967 | -1.95605 |
| C | -1.19389 | 2.05281 | -0.17280 |
| C | 0.38026 | 4.51037 | 1.90783 |
| C | -2.16905 | 5.46631 | 0.03812 |
| C | 0.45589 | 3.80760 | -4.30962 |
| C | 2.24776 | 5.06113 | 0.33573 |
| C | -2.27297 | 4.54454 | -2.24987 |
| C | -3.29556 | 0.93587 | 0.79026 |
| C | 2.90469 | 5.01023 | -0.88682 |
| C | -3.28710 | 3.22021 | -0.30227 |
| C | 0.18520 | 5.93762 | 2.43604 |
| C | -2.93867 | 1.09579 | -1.67163 |
| C | 1.26246 | 3.72923 | 2.89154 |
| C | 1.38004 | 1.72331 | -3.26325 |
| H | -0.49119 | 2.62332 | -2.78186 |
| H | 2.84792 | 4.27407 | -2.90511 |
| H | -0.59311 | 3.99986 | 1.86495 |
| H | -1.30797 | 6.09522 | -0.23237 |
| H | -2.14429 | 5.27095 | 1.11710 |
| H | -3.08694 | 6.02928 | -0.18567 |
| H | -0.02207 | 3.22949 | -5.11435 |
| H | 1.46659 | 4.07358 | -4.65449 |
| H | -0.10688 | 4.74146 | -4.17699 |
| H | 2.73211 | 5.54344 | 1.18727 |
| H | -3.16331 | 5.17059 | -2.40467 |
| H | -2.35409 | 3.66935 | -2.90541 |
| H | -1.39141 | 5.12858 | -2.54958 |
| H | -3.05028 | 1.34308 | 1.78264 |
| H | -2.90965 | -0.09118 | 0.73656 |
| H | -4.39147 | 0.90736 | 0.68045 |
| H | 3.88968 | 5.46610 | -1.00016 |
| H | -3.56618 | 3.47472 | 0.73218 |
| H | -4.19055 | 3.30607 | -0.92393 |
| H | -0.39075 | 6.57079 | 1.74951 |
| H | 1.15901 | 6.42403 | 2.59975 |

| | | | |
|---|---|---|---|
| H | -0.33648 | 5.91482 | 3.40467 |
| H | -4.02138 | 0.97635 | -1.83157 |
| H | -2.47511 | 0.09829 | -1.67516 |
| H | -2.52534 | 1.66294 | -2.51686 |
| H | 1.43295 | 2.70348 | 2.53917 |
| H | 0.77449 | 3.68055 | 3.87667 |
| H | 2.23615 | 4.22644 | 3.02223 |
| H | 0.96178 | 1.12284 | -4.08520 |
| H | 1.40859 | 1.09446 | -2.36367 |
| H | 2.41023 | 2.00305 | -3.53490 |
| Si | -0.11003 | -0.94383 | -0.59695 |
| N | 0.96236 | -3.33769 | 0.48777 |
| C | 2.69571 | -1.79606 | 0.32318 |
| C | 2.17599 | -4.17078 | 0.76854 |
| C | -0.98271 | -4.49002 | -0.50947 |
| C | -1.05019 | -3.76119 | 1.83949 |
| C | -0.36762 | -3.87700 | 0.60691 |
| C | -0.52008 | -2.97091 | 3.02716 |
| C | -2.31337 | -4.34967 | 1.95605 |
| C | 1.19389 | -2.05281 | 0.17280 |
| C | -0.38026 | -4.51037 | -1.90783 |
| C | 2.16905 | -5.46631 | -0.03812 |
| C | -0.45589 | -3.80760 | 4.30962 |
| C | -2.24776 | -5.06113 | -0.33573 |
| C | 2.27297 | -4.54454 | 2.24987 |
| C | 3.29556 | -0.93587 | -0.79026 |
| C | -2.90469 | -5.01023 | 0.88682 |
| C | 3.28710 | -3.22021 | 0.30227 |
| C | -0.18520 | -5.93762 | -2.43604 |
| C | 2.93867 | -1.09579 | 1.67163 |
| C | -1.26246 | -3.72923 | -2.89154 |
| C | -1.38004 | -1.72331 | 3.26325 |
| H | 0.49119 | -2.62332 | 2.78186 |
| H | -2.84792 | -4.27407 | 2.90511 |
| H | 0.59311 | -3.99986 | -1.86495 |
| H | 1.30797 | -6.09522 | 0.23237 |
| H | 2.14429 | -5.27095 | -1.11710 |

| | | | |
|---|---|---|---|
| H | 3.08694 | -6.02928 | 0.18567 |
| H | 0.02207 | -3.22949 | 5.11435 |
| H | -1.46659 | -4.07358 | 4.65449 |
| H | 0.10688 | -4.74146 | 4.17699 |
| H | -2.73211 | -5.54344 | -1.18727 |
| H | 3.16331 | -5.17059 | 2.40467 |
| H | 2.35409 | -3.66935 | 2.90541 |
| H | 1.39141 | -5.12858 | 2.54958 |
| H | 3.05028 | -1.34308 | -1.78264 |
| H | 2.90965 | 0.09118 | -0.73656 |
| H | 4.39147 | -0.90736 | -0.68045 |
| H | -3.88968 | -5.46610 | 1.00016 |
| H | 3.56618 | -3.47472 | -0.73218 |
| H | 4.19055 | -3.30607 | 0.92393 |
| H | 0.39075 | -6.57079 | -1.74951 |
| H | -1.15901 | -6.42403 | -2.59975 |
| H | 0.33648 | -5.91482 | -3.40467 |
| H | 4.02138 | -0.97635 | 1.83157 |
| H | 2.47511 | -0.09829 | 1.67516 |
| H | 2.52534 | -1.66294 | 2.51686 |
| H | -1.43295 | -2.70348 | -2.53917 |
| H | -0.77449 | -3.68055 | -3.87667 |
| H | -2.23615 | -4.22644 | -3.02223 |
| H | -0.96178 | -1.12284 | 4.08520 |
| H | -1.40859 | -1.09446 | 2.36367 |
| H | -2.41023 | -2.00305 | 3.53490 |

**3a**

Energy: -7438051.2239691

| | | | |
|---|---|---|---|
| Se | 5.56251 | 4.79948 | -0.09400 |
| Si | 7.16914 | 4.82230 | 1.57781 |
| N | 5.95909 | 6.98182 | 3.14908 |
| C | 7.04788 | 6.37835 | 2.74283 |
| Se | 7.19337 | 3.34178 | 3.14464 |
| C | 8.25034 | 6.92197 | 3.51531 |
| C | 4.68733 | 6.95840 | 2.43841 |
| C | 4.50238 | 7.92855 | 1.42626 |

| | | | |
|---|---|---|---|
| C | 3.69983 | 5.99402 | 2.72937 |
| C | 5.57411 | 8.92954 | 1.02121 |
| H | 6.26822 | 9.02612 | 1.85728 |
| C | 3.84947 | 4.84358 | 3.71617 |
| H | 4.87022 | 4.85999 | 4.12207 |
| C | 3.29625 | 7.92678 | 0.72479 |
| H | 3.14410 | 8.65523 | -0.07195 |
| C | 2.50480 | 6.05525 | 2.00069 |
| H | 1.72821 | 5.31722 | 2.20732 |
| C | 2.29794 | 7.00417 | 1.01146 |
| H | 1.36183 | 7.01741 | 0.45146 |
| C | 2.83288 | 4.92457 | 4.86299 |
| H | 1.80767 | 4.81322 | 4.47817 |
| H | 2.87772 | 5.87096 | 5.41544 |
| H | 3.00862 | 4.10376 | 5.57379 |
| C | 5.00451 | 10.32508 | 0.75267 |
| H | 5.82670 | 11.04731 | 0.64524 |
| H | 4.34791 | 10.66497 | 1.56761 |
| H | 4.42822 | 10.35430 | -0.18352 |
| C | 6.12648 | 7.79952 | 4.42101 |
| C | 3.67761 | 3.49261 | 3.00667 |
| H | 2.63486 | 3.34998 | 2.68274 |
| H | 3.93876 | 2.67828 | 3.69769 |
| H | 4.33530 | 3.39964 | 2.13378 |
| C | 6.38760 | 8.43056 | -0.17564 |
| H | 5.74243 | 8.15376 | -1.02266 |
| H | 6.98066 | 7.54068 | 0.07931 |
| H | 7.08806 | 9.20855 | -0.51571 |
| C | 9.37305 | 5.94377 | 3.85973 |
| H | 9.83069 | 5.51330 | 2.95759 |
| H | 9.01052 | 5.11454 | 4.47704 |
| H | 10.15248 | 6.49913 | 4.40409 |
| C | 8.85018 | 8.04528 | 2.63872 |
| H | 9.25023 | 7.63201 | 1.70211 |
| H | 9.67310 | 8.51867 | 3.19402 |
| H | 8.11951 | 8.82166 | 2.38074 |
| Se | 8.99646 | 5.11979 | 0.18306 |

| | | | |
|---|---|---|---|
| Si | 7.39437 | 5.00722 | -1.49193 |
| N | 8.74597 | 2.96497 | -3.04402 |
| C | 7.59782 | 3.42063 | -2.61077 |
| Se | 7.34504 | 6.40545 | -3.13503 |
| C | 6.44694 | 2.71359 | -3.33944 |
| C | 9.99120 | 3.02797 | -2.30282 |
| C | 10.17151 | 2.02089 | -1.32939 |
| C | 10.95378 | 4.02455 | -2.53366 |
| C | 9.12582 | 0.95335 | -1.03780 |
| H | 8.46589 | 0.87216 | -1.91344 |
| C | 10.81092 | 5.12648 | -3.56770 |
| H | 9.79740 | 5.07449 | -3.98705 |
| C | 11.34050 | 2.04934 | -0.56898 |
| H | 11.49246 | 1.29667 | 0.20529 |
| C | 12.11212 | 3.99784 | -1.74674 |
| H | 12.86969 | 4.76817 | -1.90037 |
| C | 12.30510 | 3.03053 | -0.77138 |
| H | 13.21001 | 3.04054 | -0.16212 |
| C | 11.84167 | 4.94781 | -4.69007 |
| H | 12.86023 | 5.12446 | -4.31141 |
| H | 11.82617 | 3.93306 | -5.11466 |
| H | 11.65524 | 5.66749 | -5.50104 |
| C | 9.74636 | -0.42923 | -0.81846 |
| H | 8.95409 | -1.18998 | -0.76965 |
| H | 10.44017 | -0.69948 | -1.62866 |
| H | 10.29819 | -0.47795 | 0.13131 |
| C | 8.63919 | 2.22767 | -4.36322 |
| C | 10.95246 | 6.51677 | -2.93945 |
| H | 11.97509 | 6.68566 | -2.56768 |
| H | 10.73029 | 7.28716 | -3.69222 |
| H | 10.24451 | 6.65470 | -2.11135 |
| C | 8.25960 | 1.35788 | 0.15925 |
| H | 8.87568 | 1.56151 | 1.04781 |
| H | 7.68397 | 2.27401 | -0.03814 |
| H | 7.54396 | 0.56041 | 0.41197 |
| C | 5.47681 | 3.63639 | -4.08508 |
| H | 5.00136 | 4.34937 | -3.39797 |

| | | | |
|---|---|---|---|
| H | 5.98128 | 4.22490 | -4.86135 |

## (S6) Raman spectra of 3a

Raman spectra are recorded on solid sample of **3a** which exhibit Raman bands at 1490.9 cm$^{-1}$ with a shoulder at 1475.9 cm$^{-1}$. The calculated Raman bands with the highest intensities are observed at 1605.7 and 1595.8 cm$^{-1}$. The two bands of which the later one is shouldered with the highest intensity at 1605.7 cm$^{-1}$. The Raman bands are observed in the range of 400 to 50 cm$^{-1}$ (strong lines are at around 240 cm$^{-1}$) for different polymorphs of $(SiSe_2)_n$.[S21]

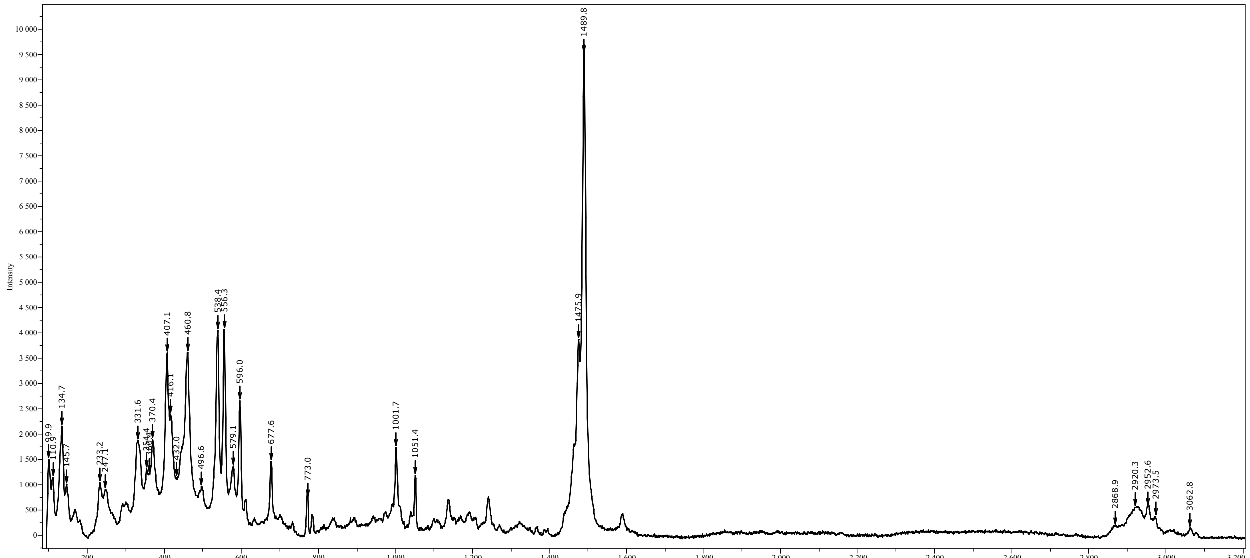

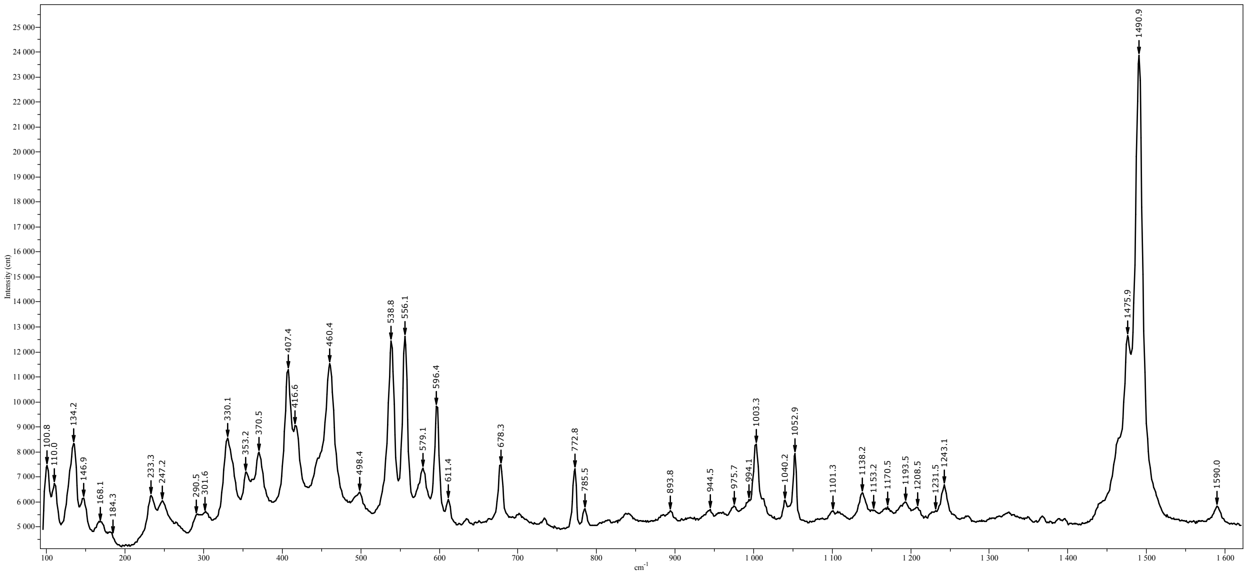
Intensity (cnt)
cm⁻¹
1490.9
1475.9
538.8
556.1
460.4
407.4
416.6
596.4
330.1
370.5
353.2
134.2
100.8
110.0
146.9
168.1
184.3
233.3
247.2
290.5
301.6
498.4
579.1
611.4
678.3
772.8
785.5
893.8
944.5
975.7
994.1
1003.3
1040.2
1052.9
1101.3
1138.2
1153.2
1170.5
1193.5
1208.5
1231.5
1243.1
1590.0

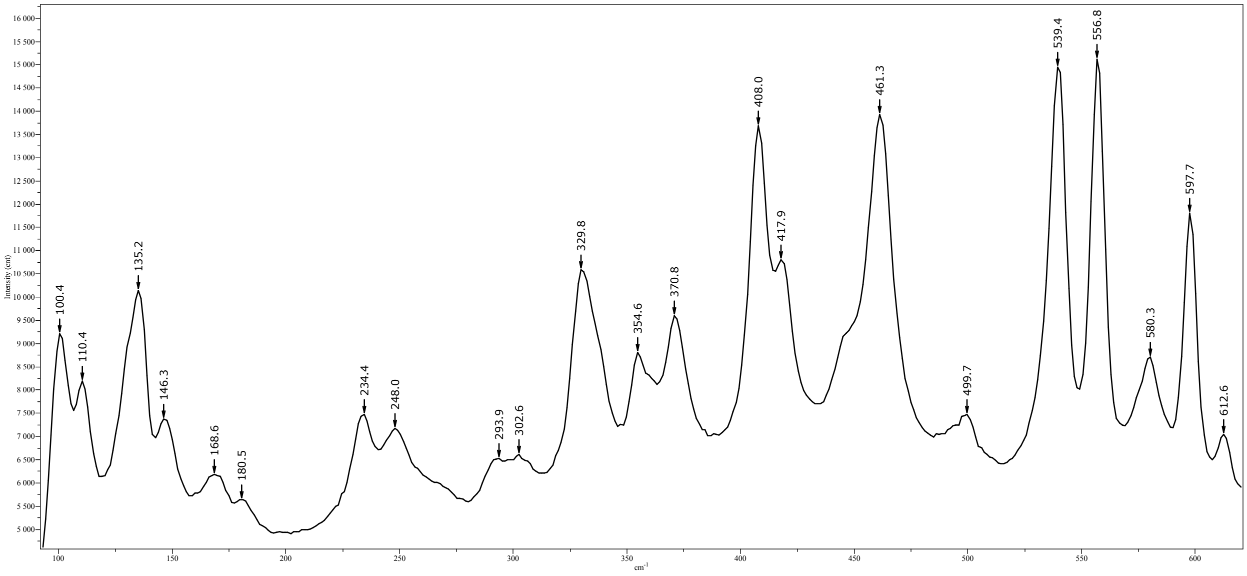


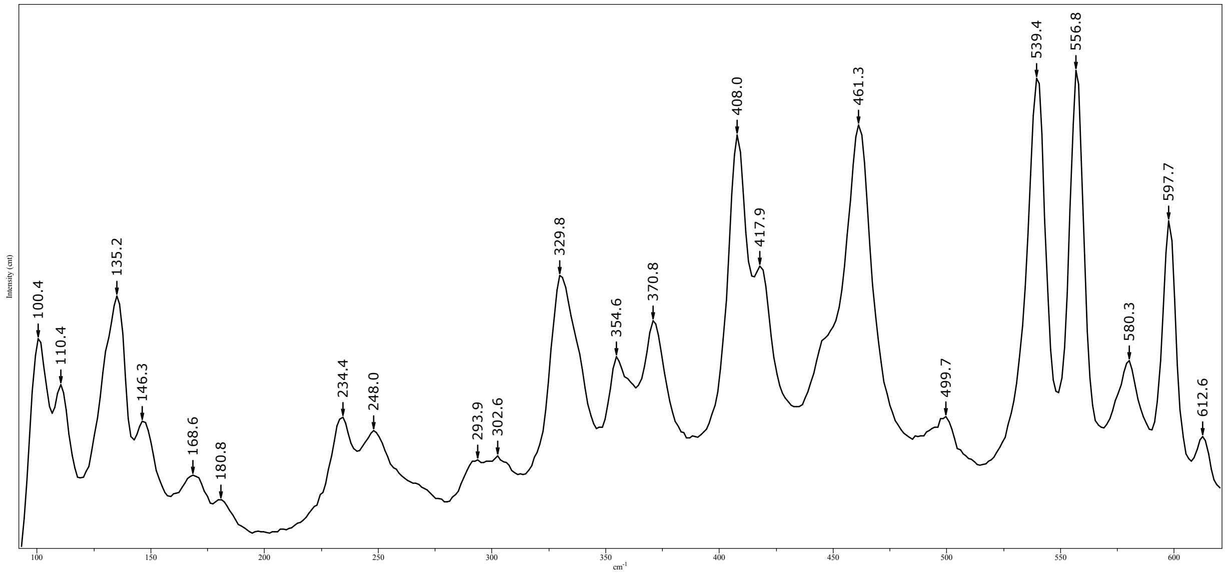


**(S7) NMR spectra**

$^{1}H$ NMR spectrum of Compound 2a.

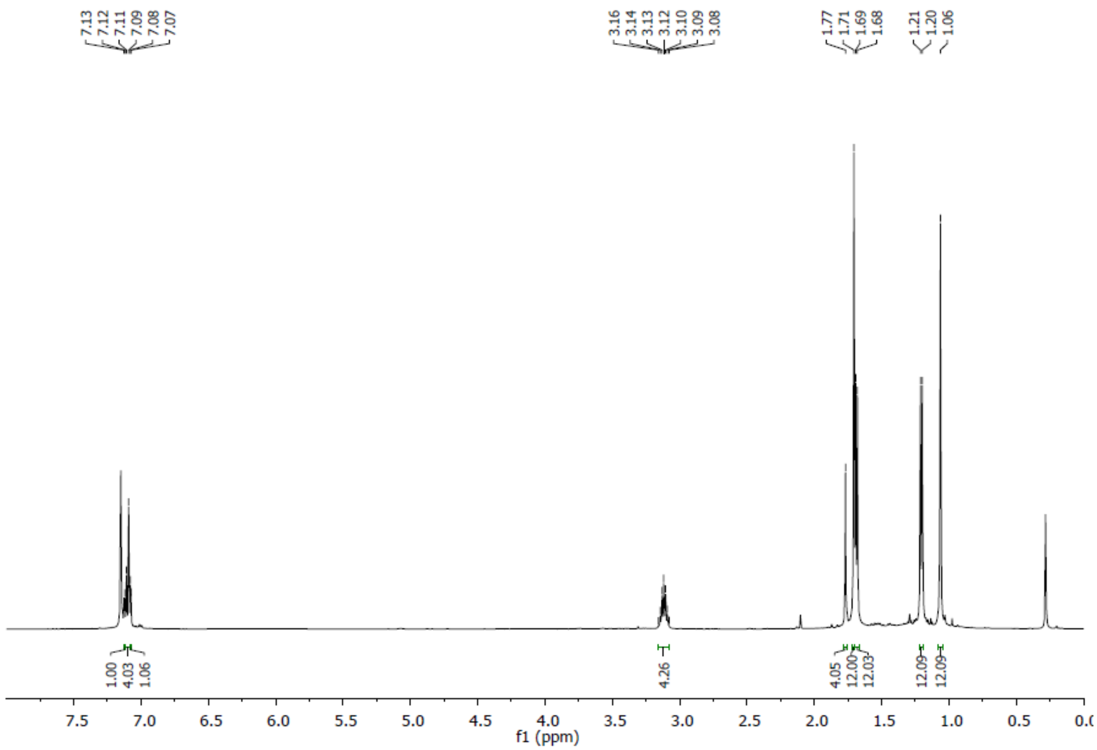

$^{13}C$ NMR spectrum of Compound 2a.

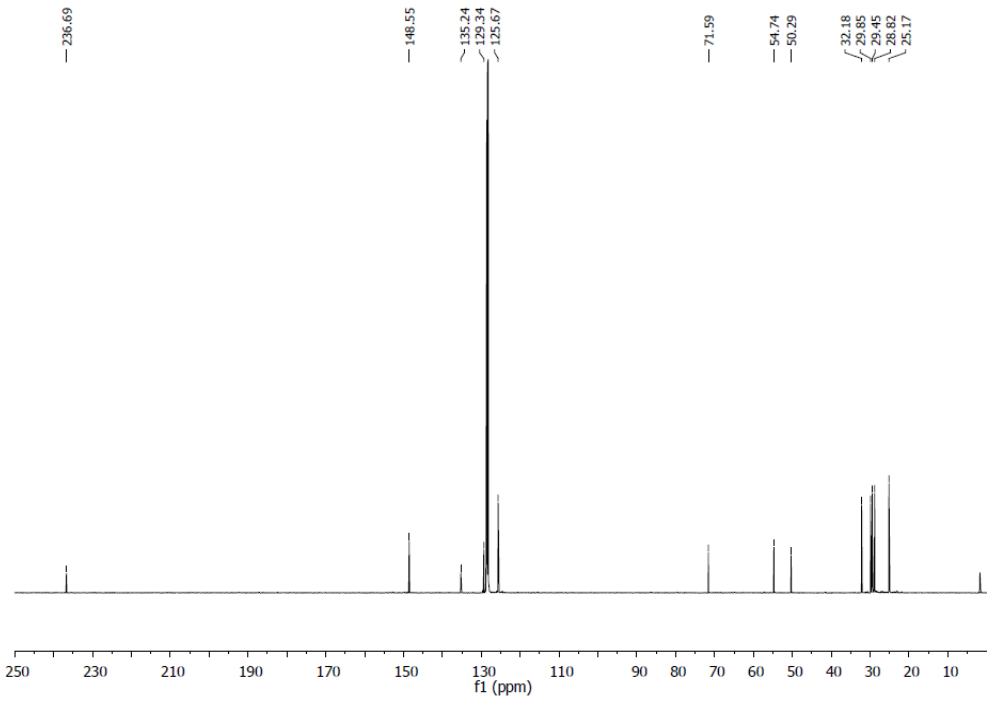

**$^{29}$Si NMR spectrum of Compound 2a.**

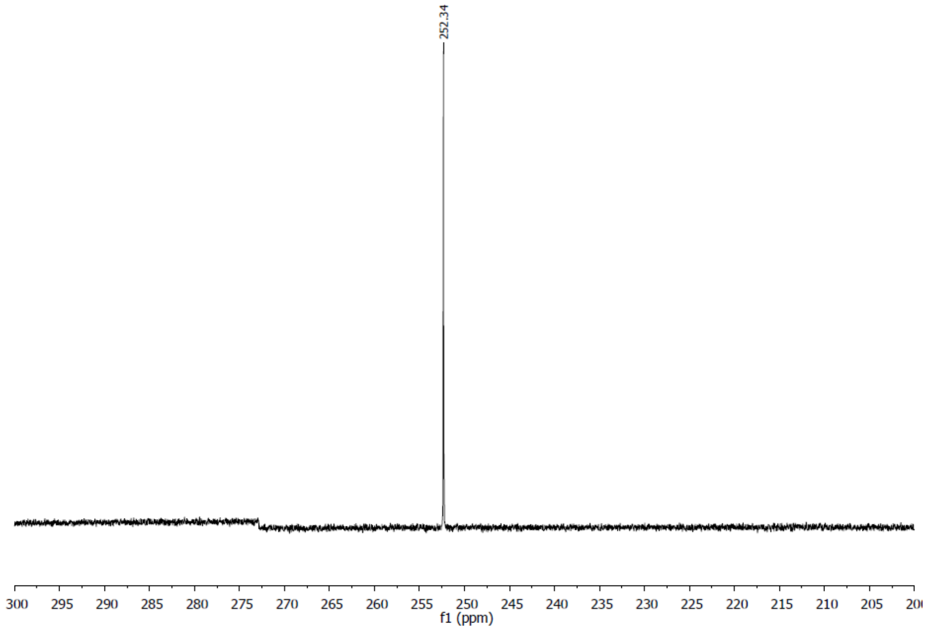


**$^{1}$H NMR spectrum of Compound 2b.**

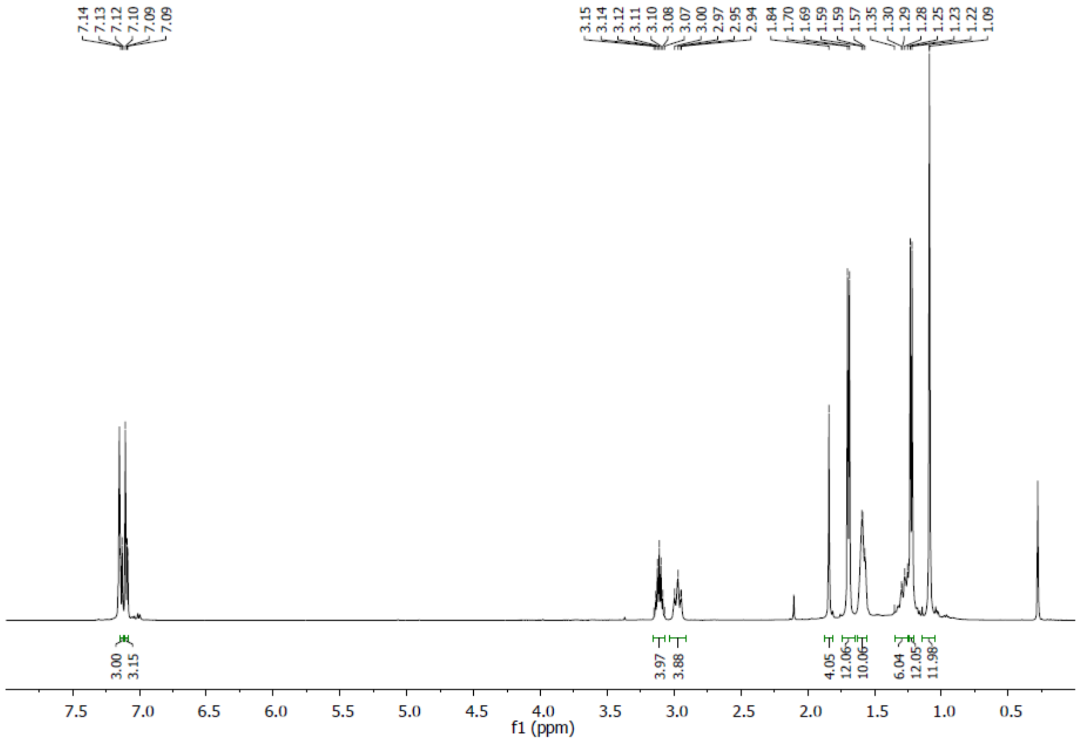

**$^{13}C$ NMR spectrum of Compound 2b.**

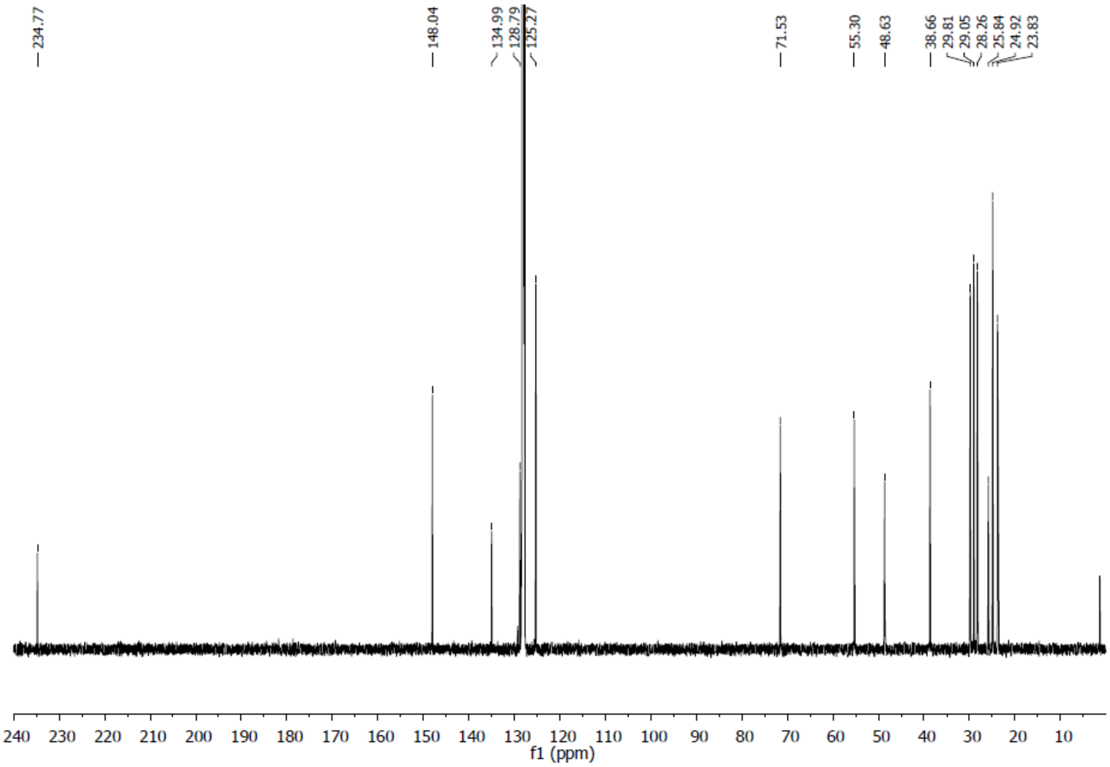


**$^{29}Si$ NMR spectrum of Compound 2b.**

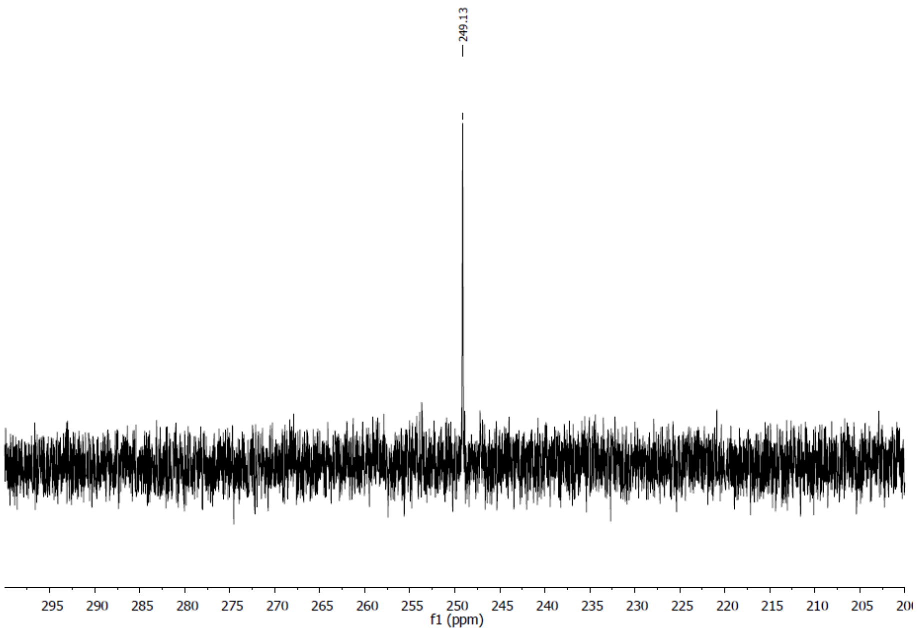

**(S8) EI-mass spectra of 3a**

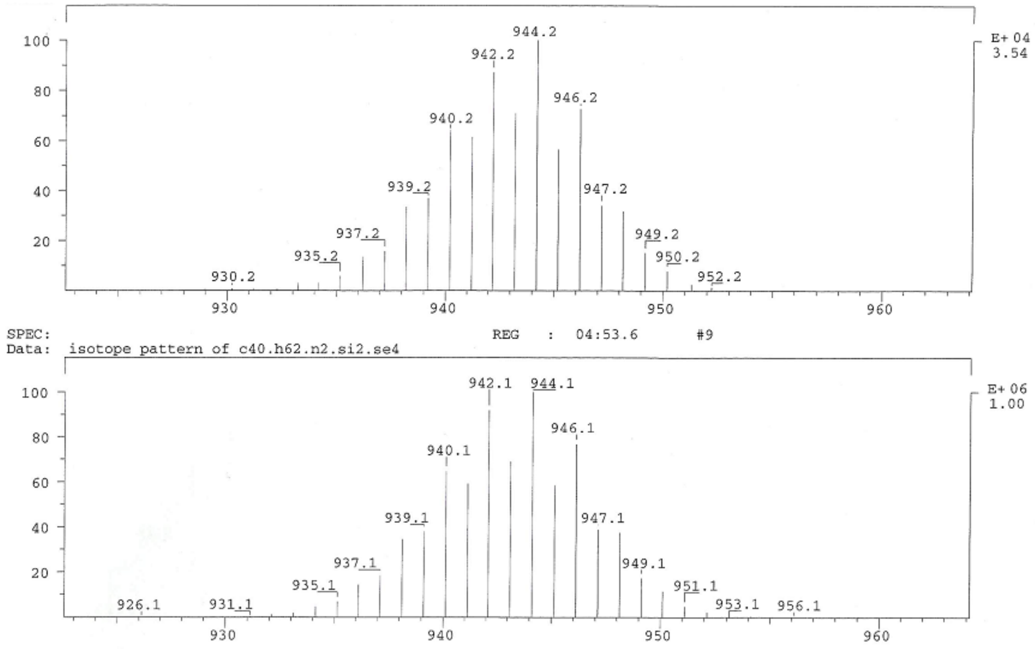


Experimental (top) and simulated (bottom) EI-MS mass spectra of **3a**: (*m/Z*(100%); 944.2).

When **3a** is heated to 300 °C it decomposes to $Me_2$-cAAC=Se which is characterized by mass spectrum (below)

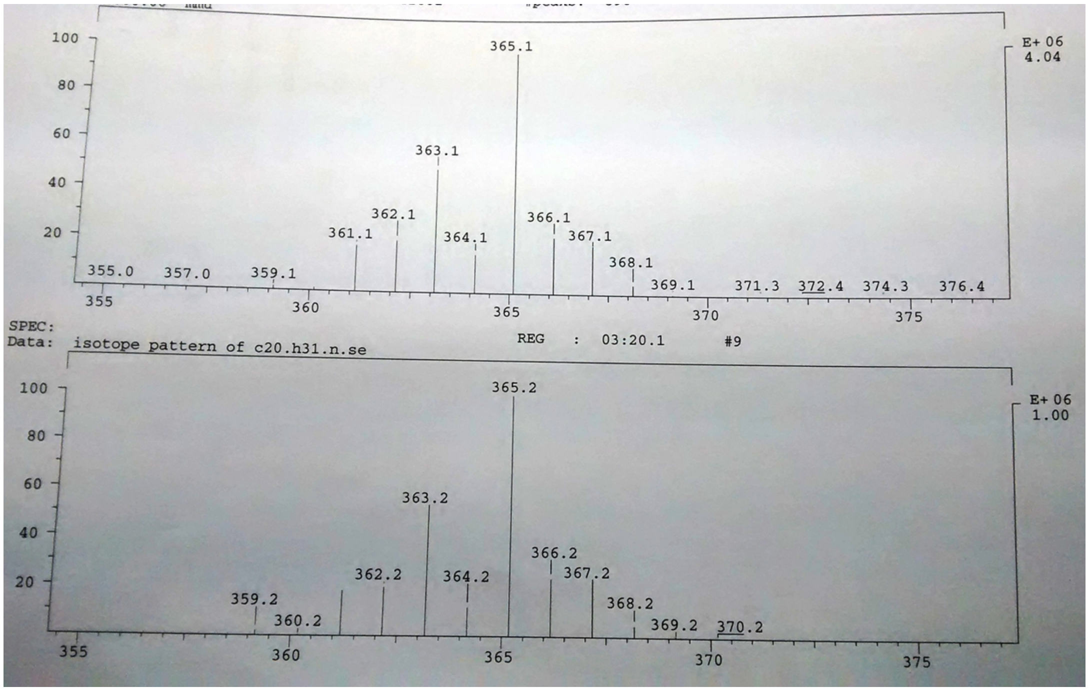


Experimental (top) and simulated (bottom) EI-MS mass spectra of $Me_2$-cAAC=Se: (*m/Z*(100%); 365.1).

## (S9) References


(S1) V. Lavallo, Y. Canac, C. Präsang, B. Donnadieu, G. Bertrand, *Angew. Chem. Int. Ed.*, 2005, *44*, 5705; *Angew. Chem.* 2005, **117**, 5851.

(S2) K. C. Mondal, H. W. Roesky, A. C. Stückl, F. Ihret, W. Kaim, B. Dittrich, B. Maity, D. Koley, *Angew. Chem. Int. Ed.*, 2013, **52**, 11804; *Angew.Chem.*, 2013, **125**, 12020.

(S3) K. C. Mondal, P. P. Samuel, H. W. Roesky, R. R. Aysin, L. A. Leites, S. Neudeck, J. Lübben, B. Dittrich, M. Hermann, G. Frenking. *J. Am. Chem. Soc.*, 2014, **136**, 8919.

(S4) Tretiakov, M.; Shermolovich, Y. G.; Singh, A. P.; Samuel, P. P.; Roesky, H. W.; Niepötter, B.; Visscher, A.; Stalke, D. *Dalton Trans.*, 2013, **42**, 12940.

(S5) K. C. Mondal, P. P. Samuel, M. Tretiakov, A. P. Singh, H. W. Roesky, A. C. Stückl, B. Niepötter, E. Carl, H. Wolf, R. Herbst-Irmer, D. Stalke, *Inorg. Chem.*, 2013, **52**, 4736.

(S6) G. M. Sheldrick, SADABS 2008/2, Göttingen, 2008.

(S7) G. M. Sheldrick, *Acta Cryst.*, 2008, **A64**, 112.

(S8) B. Dittrich, C. B. Hübschle, K. Pröpper, F. Dietrich, T. Stolper, J. Holstein, *J. Acta Cryst.*, 2013, **B69**, 91.

(S9) Volkov, A.; Macchi, P.; Farrugia, L. J.; Gatti, C.; Mallinson, P.; Richter, T.; Koritsanszky, T. XD2006 -A Computer Program Package for Multipole Refinement, Topological Analysis of Charge Densities and Evaluation of Intermolecular Energies from Experimental or Theoretical Structure Factors. University at Buffalo, NY, USA; University of Milano, Italy; University of Glasgow, UK; CNRISTM, Milano, Italy; Middle Tennessee State University, TN, USA; University of Berlin, Berlin, Germany.

(S10) Y. Wang, Y. Xie, P. Wei, R. B. King, H. F. Schaefer III, P. v. R. Schleyer, G. H. Robinson, *Science,* 2008, **321**, 1069.

(S11) K. C. Mondal, H. W. Roesky, M. C. Schwarzer, G. Frenking, B. Niepötter, H. Wolf, R. Herbst-Irmer, D. Stalke, *Angew. Chem. Int. Ed.*, 2013, **52**, 2963; *Angew.Chem.*, 2013, **125**, 3036.

(S12) (*a*) Y. Wang, Y. Xie, P. Wie, R. B. King, H. F. Schaefer, III, P. v. R. Schleyer, G. H. Robinson, *J. Am. Chem. Soc.,* 2008, **130**,14970; (*b*) O. Back, G. Kuchenbeiser, B. Donnadieu, G. Bertrand, *Angew. Chem. Int*. Ed., 2009, **48**, 5530; *Angew. Chem*., 2009, **121**, 5638.

(S13) Gaussian 09, Revision C.01, M. J. Frisch et al. Gaussian, Inc., Wallingford CT, 2009. Full reference for Gaussian09: Gaussian 09, Revision **C.01**, M. J. Frisch, G. W. Trucks, H. B. Schlegel, G. E. Scuseria, M. A. Robb, J. R. Cheeseman, G. Scalmani, V. Barone, B. Mennucci, G. A. Petersson, G. H. Nakatsuji, M. Caricato, X. Li, H. P. Hratchian, A. F. Izmaylov, J. Bloino, G. Zheng, J. L. Sonnenberg, M. Hada, M. Ehara, K. Toyota, R. Fukuda, J. Hasegawa, M. Ishida, T. Nakajima, Y. Honda, O. Kitao, H. Nakai, T. Vreven, Jr. J. A. Montgomery, J. E. Peralta, F. Ogliaro, M. Bearpark, J. J. Heyd, E. Brothers, K. N. Kudin, V. N. Staroverov, R. Kobayashi, J. Normand, K. Raghavachari, A. Rendell, J. C. Burant, S. S. Iyengar, J. Tomasi, M. Cossi, N. Rega, J. M. Millam, M. Klene, J. E. Knox, J. B. Cross, V. Bakken, C. Adamo, J. Jaramillo, R. Gomperts, R. E. Stratmann, O. Yazyev, A. J. Austin, R. Cammi, C. Pomelli, J. W. Ochterski,

R. L. Martin, K. Morokuma, V. G. Zakrzewski, G. A. Voth, P. Salvador, J. J. Dannenberg, S. Dapprich, A. D. Daniels, Ö. Farkas, J. B. Foresman, J. V. Ortiz, J. Cioslowski, D. J. Fox, Gaussian, Inc., Wallingford CT, **2010**.

(S14) Y. Zhao, N. E. Schultz, D. G. Truhlar, *J. Chem. Theory and Comput*., 2006, **2**, 364-382.

(S15) A. Schäfer, H. Horn, R. Ahlrichs, *J. Chem. Phys*., 1992, **97**, 2571-2577.

(S16) A. Schäfer, C. Huber, R. Ahlrichs, *J. Chem. Phys*., 1994, **100**, 5829-5835.

(S17) A. V. Marenich, C. J. Cramer, D. G. Truhlar, *J. Phys. Chem. B,* 2009, **113**, 6378–6396.

(S18) AIMAll (Version 13.11.04, Standard), Copyright © 1997-2013, T. A. Keith http://aim.tkgristmill.com

(S19) R. F. W. Bader, *Chem. Rev*., 1991, **94**, 893-928

(S20) http://www.chemcraftprog.com.

(S21) A. Pradel, V. Michel-Lledos, M. Ribes, H. Eckert, *Chem. Mater*., 1993, **5**, 377.
